# Targeting Hippocampal PTEN Suppresses Ferroptosis and Rescues Cognitive Decline in Alzheimer's Disease via Dual AKT/GSK3β/Nrf2 and AKT/STAT3 Axes

**DOI:** 10.1002/advs.76989

**Published:** 2026-08-03

**Authors:** Da‐Wei Wang, Meng‐Meng Liu, Yu‐Chen Zhao, Jia‐Yi Li, Wen Li, Xin Yu

**Affiliations:** ^1^ Key Laboratory of Medical Cell Biology of Ministry of Education Key Laboratory of Major Chronic Diseases of Nervous System of Liaoning Province Health Sciences Institute of China Medical University Shenyang China; ^2^ Department of Neurology Second Affiliated Hospital Army Medical University (Third Military Medical University) Chongqing China; ^3^ Key Laboratory of Major Chronic Diseases of Nervous System of Liaoning Province Department of Physical Education Institute of China Medical University Shenyang China; ^4^ Laboratory of Research in Parkinson's Disease and Related Disorders Liaoning Provincial Key Laboratory of Major Neurological Diseases Health Sciences Institute of China Medical University Shenyang China

**Keywords:** alzheimer's disease, ferroptosis, glutathione peroxidase 4, nuclear factor erythroid 2‐related factor 2, phosphatase and tensin homolog, signal transducer and activator of transcription 3

## Abstract

Elevated phosphatase and tensin homolog (PTEN) expression is observed in Alzheimer's disease (AD) brain, yet the precise mechanism through which PTEN contributes to AD progression remains undefined. This study provides the direct evidence that PTEN promotes neurodegeneration by driving neuronal ferroptosis. Using APP/PS1 transgenic mice with hippocampal‐specific PTEN knockdown mediated by adeno‐associated virus (AAV), we demonstrated that downregulation of PTEN substantially ameliorates cognitive dysfunction and neuronal loss. Mechanistically, PTEN silencing upregulated glutathione peroxidase 4 (GPX4), inhibiting lipid peroxidation and ferroptosis. We identified a dual‐signaling framework through which PTEN regulates GPX4 expression. PTEN reduction activates the PI3K/AKT axis, which drives GSK3β phosphorylation and facilitates nuclear translocation of nuclear factor erythroid 2‐related factor 2 (Nrf2). Concurrently, PTEN knockdown induces phosphorylation and nuclear translocation of signal transducer and activator of transcription 3 (STAT3). Both Nrf2 and STAT3 act as transcriptional activators of GPX4, establishing two convergent axes: PTEN/AKT/GSK3β/Nrf2/GPX4 and PTEN/AKT/STAT3/GPX4. These pathways cooperatively upregulate GPX4 expression, thereby attenuating lipid peroxidation and inhibiting ferroptosis. Importantly, PTEN knockdown restored redox homeostasis by bolstering cellular antioxidant defenses. Our findings reveal a novel PTEN‐regulated ferroptotic pathway in AD pathogenesis and highlight PTEN as a promising therapeutic target for AD.

## Graphical abstract

PTEN knockdown activates AKT phosphorylation, promoting Nrf2 nuclear translocation and STAT3 activation, which upregulates GPX4 and antioxidant enzymes HO‐1, SOD1, SOD2, and NQO1. These coordinated changes reduce lipid peroxidation and ROS, inhibit ferroptosis, and ultimately ameliorate cognitive impairment in APP/PS1 transgenic mice, highlighting a potential therapeutic axis for neurodegenerative disease.

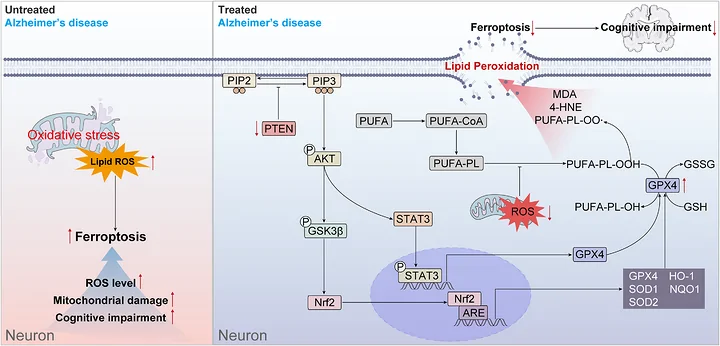

## 1 Introduction

The key neuropathological features of Alzheimer's disease (AD) include β‐amyloid protein (Aβ) deposition, hyperphosphorylated tau accumulation, chronic neuroinflammation, and progressive neuronal loss [1]. Neuronal loss constitutes the primary structural basis of irreversible cognitive impairment and closely correlates with synaptic degeneration and cognitive deficits in patients [2]. Apoptosis, evidenced by caspase activation, mitochondrial dysfunction, and deoxyribonucleic acid (DNA) fragmentation, has long been regarded as the classical mechanism of neuronal death in AD [3]. Necroptosis, a regulated form of cell death mediated by receptor‐interacting protein kinase 1 (RIPK1), receptor‐interacting protein kinase 3 (RIPK3), and mixed lineage kinase domain‐like protein (MLKL), has emerged as an alternative contributor to AD‐related neurodegeneration [4]. However, emerging research suggests that ferroptosis also exerts a substantial influence on AD progression [5]. Increasing evidence indicates that iron dyshomeostasis and ferroptosis‐related lipid peroxidation are not only involved in classical neurodegenerative mechanisms but may also represent promising therapeutic targets for cognitive decline and dementia [6]. Despite its growing importance, the specific molecular events underlying neuronal ferroptosis in AD remain insufficiently defined.

Ferroptosis has emerged as a distinct and increasingly recognized form of regulated cell death that contributes to neurodegenerative processes, including AD. Unlike classical cell death pathways, ferroptosis is characterized by iron‐dependent lipid peroxidation and specific mitochondrial abnormalities, such as membrane compaction and cristae loss [7]. Accumulating evidence indicates that dysregulated iron metabolism and excessive lipid peroxidation, which are hallmarks of ferroptosis, are prominent features of the AD brain and closely associated with neuronal degeneration [8, 9]. Glutathione peroxidase 4 (GPX4) plays a pivotal role in controlling ferroptosis by detoxifying lipid peroxides and maintaining neuronal redox homeostasis [10]. Its expression is regulated by transcription factors such as nuclear factor erythroid 2‐related factor 2 (Nrf2) and signal transducer and activator of transcription 3 (STAT3). For instance, Nrf2 has been shown to transcriptionally upregulate GPX4 in acute myeloid leukemia cells, identifying GPX4 as a direct NRF2 target that attenuates ferroptosis [11]. Importantly, Nrf2 activity is tightly regulated by the AKT/GSK3β axis: AKT phosphorylation inhibits GSK3β, thereby preventing Nrf2 nuclear export and degradation, and promoting its stabilization and nuclear translocation. This pathway is a key upstream regulator of antioxidant responses and ferroptosis resistance [12]. Additionally, phosphorylated STAT3 translocates to the nucleus to enhance GPX4 expression, mitigating ferroptosis and oxidative damage in ischemia‐reperfusion injury [13]. However, the specific role of the STAT3/GPX4 axis in regulating ferroptosis in AD remains unclear. Several studies in AD models have shown that GPX4 overexpression attenuates neuronal ferroptosis, reduces oxidative damage and preserves cognitive function [14, 15]. These findings underscore the critical roles of Nrf2/GPX4 and STAT3/GPX4 signaling pathways in AD pathophysiology.

Phosphatase and tensin homolog (PTEN) is a well‐characterized tumor suppressor that regulates cellular homeostasis, including cell growth, survival, and apoptosis [16]. Elevated PTEN levels have been observed in the brains of AD model mice, and PTEN silencing has been shown to have protective effects against neuronal apoptosis [17, 18]. While PTEN's role in apoptosis is well established, its potential involvement in regulating ferroptosis in AD remains unknown. Notably, studies in non‐neural contexts, such as liver injury models, demonstrate that PTEN inhibition can upregulate GPX4 and suppress ferroptosis [19]. Furthermore, PTEN suppression provides neuroprotection by reducing Aβ‐induced oxidative stress and neurotoxicity in SH‐SY5Y cells [20]. And in APP/PS1 mice, PTEN downregulation improves synaptic function and cognitive performance, while attenuating pathological tau phosphorylation [21, 22, 23]. Taken together, these findings suggest that reducing PTEN expression may ameliorate AD pathology by influencing GPX4, providing a potential therapeutic strategy for AD.

In this study, we hypothesize that PTEN promotes neuronal ferroptosis in AD by suppressing the AKT pathway, thereby impairing the activation of Nrf2 and STAT3 and leading to downregulation of GPX4. To test this hypothesis, we employed both APP/PS1 transgenic mice and in vitro neuronal models. Our results demonstrate that PTEN downregulation suppresses ferroptosis by enhancing AKT‐mediated phosphorylation of GSK3β (p‐GSK3β) and STAT3 (p‐STAT3), thereby enhancing Nrf2 nuclear translocation and upregulating GPX4 expression. These findings collectively support PTEN modulation as a potential therapeutic strategy for alleviating AD‐related cognitive impairment.

## 2 Results

### 2.1 Elevated PTEN Expression in AD Brain

We first performed bioinformatic analyses comparing PTEN expression between healthy individuals and AD patients. Gene intersection analysis identified 40 overlapping genes between 200 AD‐related genes (GeneCards) and 484 ferroptosis‐related genes (FerrDb), with PTEN being a key candidate (Figure 1A). Network analysis of these overlapping genes further highlighted their functional associations (Figure 1B). Transcriptomic data from the GSE122063 dataset (GEO database) revealed a significant up‐regulation of PTEN in AD patients compared to controls (Figure 1C). To validate these findings, we assessed PTEN expression in AD patient brains and APP/PS1 transgenic mouse brains. Immunofluorescence showed markedly increased PTEN levels in the cortical regions of AD patients relative to age‐matched controls (Figure 1D). Immunohistochemical staining further confirmed increased PTEN levels in cortical and hippocampal tissues of APP/PS1 mice (Figure 1E,F; Figure S1). Western blot analysis further confirmed increased PTEN protein expression in the brains of APP/PS1 mice at different ages compared with wild‐type (WT) mice matched for age (Figure 1G,H). Collectively, these multimodal analyses establish PTEN overexpression as a consistent feature of AD pathophysiology.

**FIGURE 1:**
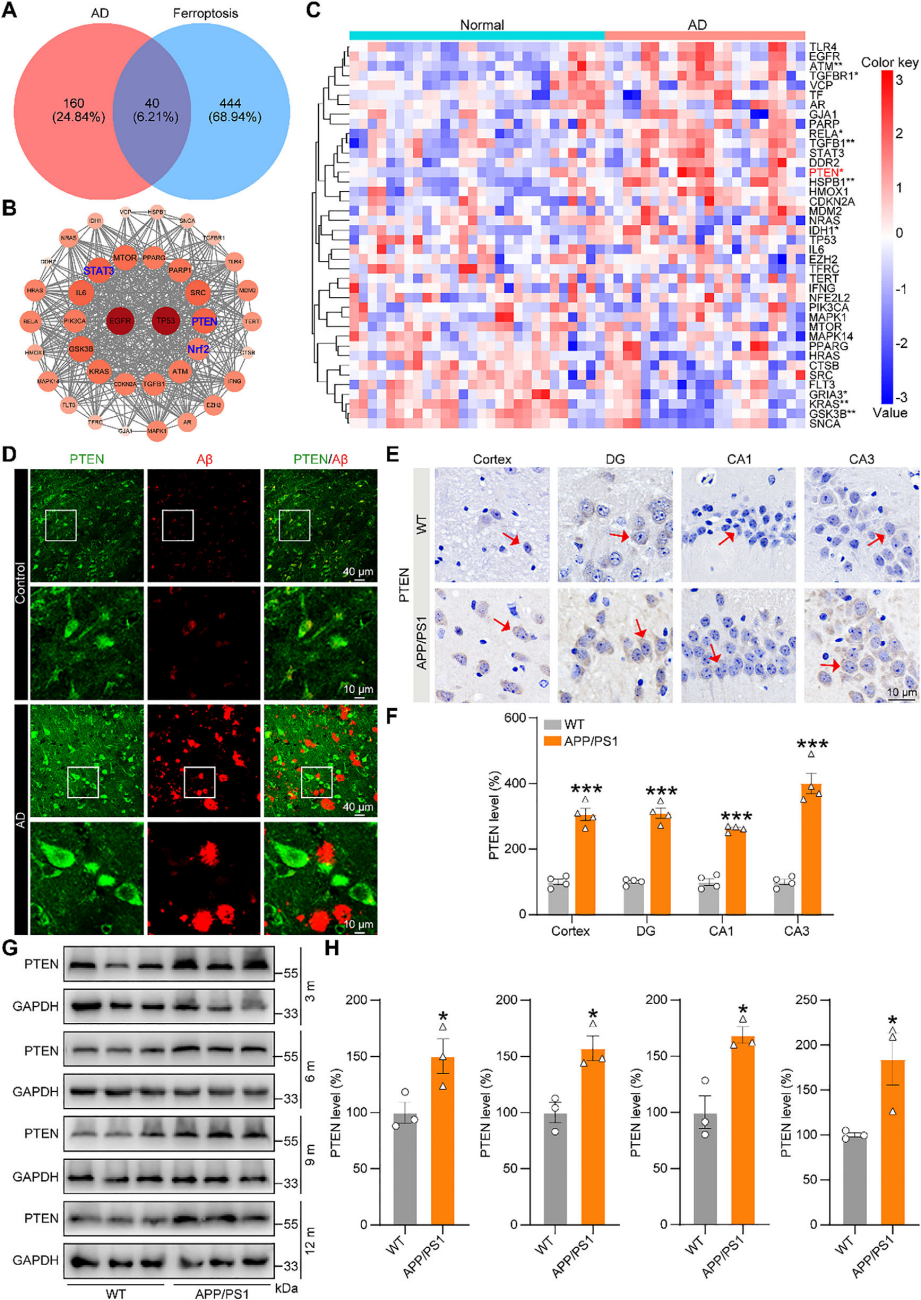
Elevated levels of PTEN in AD brain. (A) The intersection of AD genes and ferroptosis genes is presented by the Venn diagram. (B) PPI network of intersecting genes between AD and ferroptosis based on Cytoscape plug‐in MCODE analysis. (C) Heatmap showed the expression of intersecting genes in the brain of AD and control. (D) Representative images showing double immunofluorescent staining for PTEN (green) and Aβ (red) protein in the brain of AD patients. Scale bars, 40 µm and 10 µm. (E, F) Representative immunohistochemical photographs of PTEN in different brain regions of APP/PS1 mice and WT mice. Scale bars, 10 µm. *n* = 4. (G, H) Western blot showing the expression levels of PTEN in the brains of APP/PS1 mice and WT mice. *n* = 3. *p < 0.05, **p < 0.01, ***p < 0.001.

### 2.2 PTEN Knockdown Ameliorated Cognitive Deficits in APP/PS1 Mice

To investigate the influence of hippocampal PTEN knockdown in APP/PS1 transgenic mice, we generated AAV‐shPTEN and control AAV‐Scramble (Figure 2A). Initial stereotaxic delivery of GFP‐tagged AAV‐shPTEN into 4‐month‐old APP/PS1 mice confirmed successful hippocampal and cortical transduction by fluorescence imaging (Figure 2B,C; Figure S2A). In subsequent experiments, non‐fluorescent AAV‐shPTEN or Scramble was injected bilaterally into the hippocampus. Cognitive function was assessed using the MWM. During visible platform trials, no differences in escape latency or path length were observed between shPTEN‐treated and control mice, confirming preserved vision and motor function (Figure 2D). In contrast, hidden platform trials revealed significantly reduced escape latencies in shPTEN‐treated mice on days 4–5 (Figure 2E,G). Spatial exploration trials also revealed increased platform crossings in the shPTEN group compared to controls (Figure 2F,H), indicating improved spatial memory performance. Nesting behavior, a genetically programmed social activity that declines with age in APP/PS1 mice, was also assessed. PTEN knockdown significantly attenuated the age‐related decline in nesting ability (Figure 2I,J), suggesting improved cognitive and behavioral function.

**FIGURE 2:**
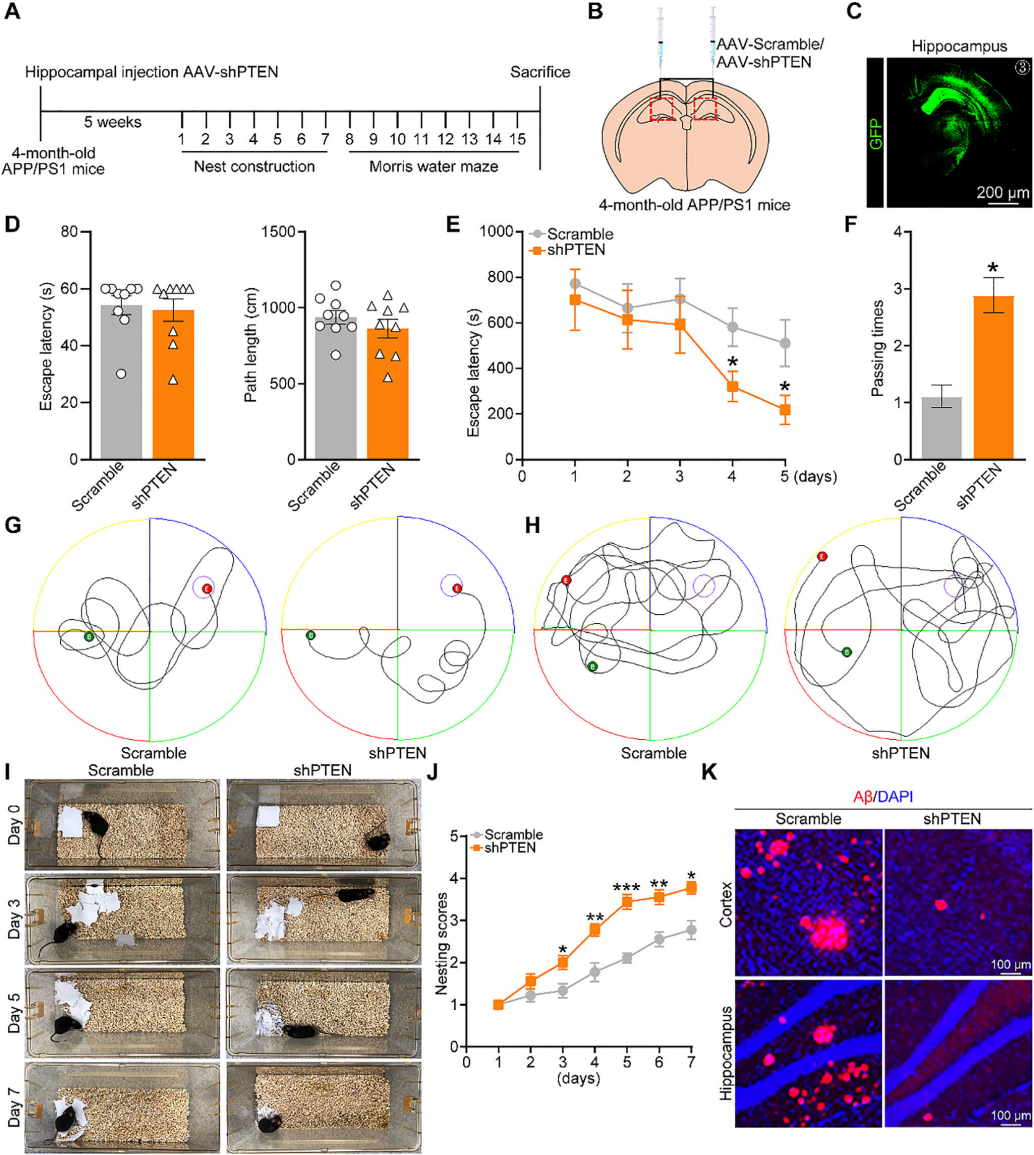
PTEN knockdown ameliorated cognitive dysfunction in APP/PS1 mice. (A) Schematic of the time course in this study. (B) Schematic of stereotactic injection of AAV‐shPTEN (or AAV‐Scramble) into the brain. (C) Fluorescence distribution in the hippocampus of APP/PS1 mice after hippocampal injection of GFP‐labelled AAV‐shPTEN for 5 weeks. Scale bar, 200 µm. (D) Escape latency and path length of the shPTEN or Scramble group to find the visible platform during the visible platform test. (E, G) The escape latency and representative path lengths of mice on days 4 and 5 to find the hidden platform during the hidden platform test. (F, H) The number and movement trajectories of mice crossing the original platform during the spatial exploration trials. (I, J) Representative graphs of the nesting experiment. *n* = 9. (K) Immunofluorescence labeling of Aβ plaques in the cortex and hippocampus of APP/PS1 mouse brains. Scale bar, 100 µm. *p < 0.05, **p < 0.01, ***p < 0.001.

Consistently, immunofluorescence analysis revealed a reduced Aβ plaque burden in both the cortex and hippocampus of shPTEN‐treated mice (Figure 2K; Figure S2B). Given the relatively limited tau pathology in APP/PS1 mice, we further assessed phosphorylated tau by co‐immunostaining with NeuN. A modest reduction in p‐Tau (Thr231) was observed following PTEN knockdown, whereas p‐Tau (Ser396) remained unchanged, suggesting a limited impact on tau pathology under the present conditions (Figure S3A, B). To further assess synaptic alterations, we examined PSD95 and Synapsin expression. PTEN knockdown significantly increased PSD95 levels, while Synapsin showed no significant change (Figure S2C), indicating a selective improvement in synaptic integrity. Taken together, these results demonstrate that PTEN reduction in the hippocampus can restore cognitive function in APP/PS1 mice.

### 2.3 PTEN Knockdown Suppressed Ferroptosis via GPX4 Upregulation in APP/PS1 Mice

To validate the cell‐type specificity of AAV‐shPTEN mediated knockdown, co‐immunofluorescence staining of PTEN with NeuN, GFAP, and Iba1 was performed. PTEN was predominantly expressed in neurons, and its expression was markedly reduced in NeuN‐positive cells following shPTEN treatment, whereas no obvious changes were observed in GFAP‐positive astrocytes or Iba1‐positive microglia (Figure 3A; Figure S4A, B), indicating neuron‐specific knockdown. Consistently, Western blot analysis confirmed a significant reduction of PTEN protein levels in shPTEN‐treated mice compared with Scramble controls (Figure 3B, C). We then assessed the levels of lipid peroxidation and mitochondrial morphology, the typical phenotypes of ferroptosis. Ultrastructural analysis by TEM further revealed attenuated ferroptotic features in shPTEN‐treated mice, including preserved mitochondrial morphology with reduced shrinkage and cristae loss (Figure 3D). Western blot results showed that shPTEN treatment significantly reduced MDA and 4‐HNE levels compared to Scramble‐treated mice (Figure 3E,F). Spearman correlation analysis showed that platform crossing times at day 8 were positively correlated with GPX4 levels and negatively correlated with 4‐HNE and MDA, indicating a relationship between reduced ferroptosis and improved cognitive performance (Figure S5A‐C). To delineate the molecular mechanism by which PTEN knockdown suppresses ferroptosis, we systematically examined key regulators of ferroptosis. Notably, GPX4, a central enzyme that detoxifies lipid peroxides through glutathione‐dependent reduction, was significantly upregulated in the shPTEN group (Figure 3G,H). In contrast, other established ferroptosis‐related markers, including FSP1, ACSL4, DMT1, and TFR1, remained unchanged (Figure 3G,H). In addition, brain iron assays showed no significant difference in ferrous iron levels between groups (Figure S6).

**FIGURE 3:**
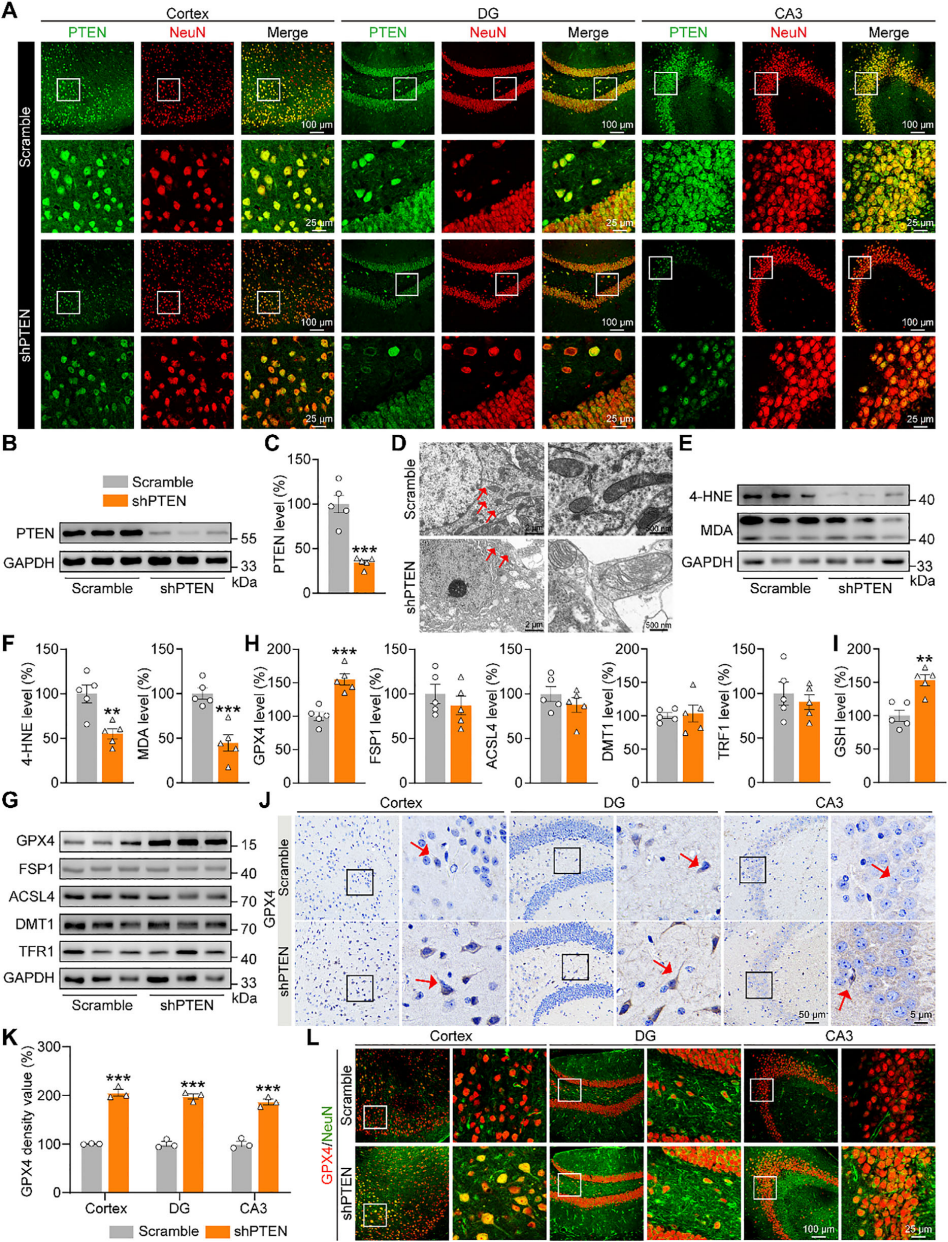
PTEN knockdown inhibited ferroptosis by upregulating GPX4 expression in APP/PS1 mouse brain. (A) Representative immunofluorescence images of PTEN/NeuN staining in different brain regions of APP/PS1 transgenic mice. Scale bars, 100 µm and 25 µm. (B, C, E‐H) Protein levels of PTEN, 4‐HNE and MDA were examined using Western blot. *n* = 5. (D) Morphological structures of intracellular mitochondria (red arrow) were observed by TEM. Scale bars, 2 µm and 500 nm. (F, G) Western blot showing the protein levels of GPX4, FSP1, ACSL4, DMT1 and TFR1 in the APP/PS1 mouse brain. *n* = 5. (I) GSH levels in the mouse hippocampus were measured using the GSH Assay Kit. *n* = 5. (J, K) Immunohistochemical staining with anti‐GPX4 antibody showing the localization of GPX4 in the cortex, DG and CA3 regions of the hippocampus in APP/PS1 mice. (L) Immunofluorescence staining of GPX4/NeuN showing GPX4 localization in the cortex, DG, and CA3 regions of APP/PS1 mice. Scale bars, 50 µm and 5 µm. *n* = 3. **p < 0.01, ***p < 0.001.

Consistent with enhanced GPX4 activity, levels of GSH were also elevated in shPTEN‐treated mice (Figure 3I), further supporting a selective reinforcement of the GPX4‐mediated antioxidant defense system against ferroptosis. Immunohistochemistry confirmed increased GPX4 expression in cortical and hippocampal regions of shPTEN‐treated mice (Figure 3J,K). To determine the cellular specificity of GPX4 upregulation, we performed co‐immunofluorescence staining of GPX4 with NeuN, GFAP, and Iba1. The results showed that increased GPX4 expression was predominantly observed in neurons, with minimal changes in astrocytes and microglia (Figure 3L; Figures S7 and S8A,B). To further examine whether other regulated cell death pathways might be involved, we evaluated representative markers of apoptosis, inflammasome activation, and necroptosis in vivo. Western blot analysis revealed no significant differences in the expression levels of apoptosis‐related proteins (Cleaved Caspase‐3, Bcl‐2, and Bax), inflammasome‐associated proteins (Cleaved Caspase‐1 and NLRP3), or necroptosis markers (RIPK1, RIPK3, and p‐MLKL) between groups (Figure S4C‐F). These results do not support a major contribution of apoptosis, pyroptosis, or necroptosis under the present experimental conditions, and instead are consistent with a predominant involvement of ferroptosis‐related mechanisms.

These results demonstrated that PTEN knockdown selectively enhanced GPX4‐mediated antioxidant defense, thereby suppressing neuronal ferroptosis in APP/PS1 mice.

### 2.4 PTEN Knockdown Activated the AKT/GSK3β/Nrf2 and AKT/STAT3 Pathways in APP/PS1 Mice

To elucidate the mechanism of ferroptosis inhibition by PTEN knockdown, we performed RNA sequencing on hippocampal tissue from APP/PS1 mice. Comparative analysis identified 1,984 differentially Expressed Genes (DEGs), including 56 ferroptosis‐related genes, between shPTEN and control groups (Figure 4A). Protein‐protein interaction network and heatmap analyses of these genes revealed significant downregulation of PTEN, upregulation of Nrf2 and STAT3 in the shPTEN group (Figure 4B,C). KEGG pathway analysis further implicated PI3K‐AKT signaling is prominently involved in regulation (Figure 4D).

**FIGURE 4:**
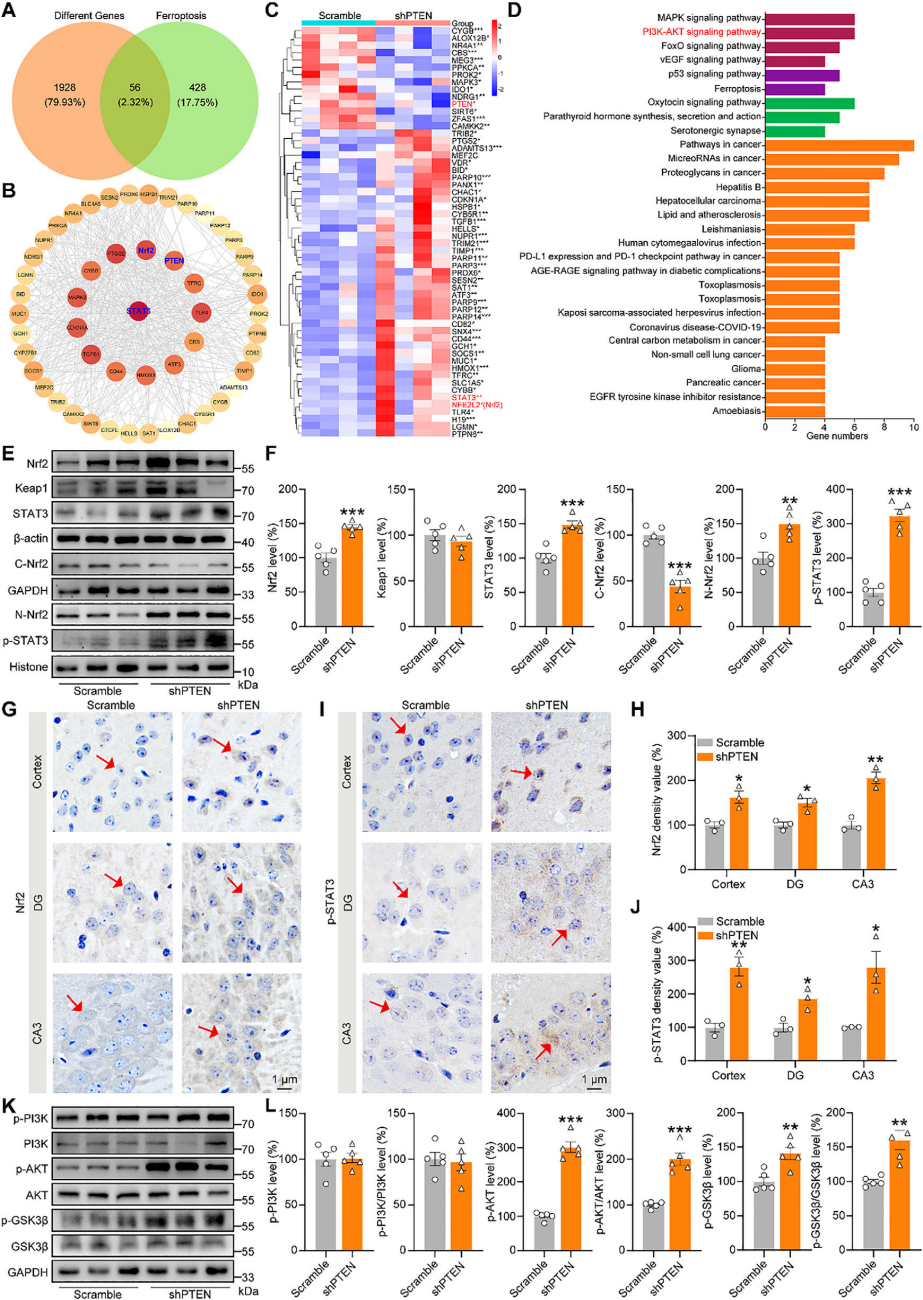
PTEN knockdown regulated the AKT/Nrf2 pathway in APP/PS1 mouse brain. (A) Venn diagram of intersecting genes, showing significant enrichment of overlapping genes compared with random expectation. (B) PPI network of intersecting genes using Cytoscape software. (C) Heatmap showing the expression of intersecting genes between Scramble and shPTEN groups. (D) Correlations between intersecting genes and pathway through KEGG enrichment analysis. (E, F) Protein expression levels of Nrf2, Keap1, STAT3, C‐Nrf2, N‐Nrf2 and p‐STAT3 were measured using immunoblotting. *n* = 5. (G‐J) Immunohistochemical staining with Nrf2 antibody or p‐STAT3 antibody showing the localization of Nrf2 in the cortex, DG and CA3 regions in APP/PS1 mouse brain. Scale bars, 1 µm. *n* = 3. (K, L) Protein levels of p‐PI3K, PI3K, p‐AKT, AKT, p‐GSK3β and GSK3β were measured by Western blot. *n* = 5. *p < 0.05, **p < 0.01, ***p < 0.001.

Given the role of PTEN as a PI3K/AKT pathway inhibitor and the established function of Nrf2 and p‐STAT3 in regulating GPX4, we hypothesized that PTEN knockdown would increase GPX4 expression via AKT/Nrf2 and AKT/STAT3 activation. Immunoblotting revealed that shPTEN‐treated mice exhibited increased Nrf2 protein levels without changes in its negative regulator Keap1, along with a significant elevation in p‐STAT3 levels (Figure 4E,F). Subcellular localization analysis demonstrated a reduction in cytoplasmic Nrf2 accompanied by enhanced nuclear accumulation in shPTEN‐treated mice, indicative of Nrf2 activation (Figure 4G,H; Figure S9A). Furthermore, shPTEN treatment markedly increased p‐STAT3 expression in the nuclear compartment (Figure 4I,J; Figure S9B). Importantly, as shown in Figure 4K,L, PTEN knockdown selectively increased AKT phosphorylation without affecting PI3K activation, and enhanced GSK3β phosphorylation, which inactivates GSK3β and thereby promotes Nrf2 activation.

In summary, these results indicated that PTEN reduction activated the AKT/GSK3β/Nrf2 and AKT/STAT3 signaling pathways, thereby upregulating GPX4 expression and suppressing ferroptosis in APP/PS1 mice.

### 2.5 PTEN Knockdown Reduced Oxidative Stress in APP/PS1 Mouse Brain

Oxidative stress‐induced lipid peroxidation is a major contributor to ferroptosis in AD brain. Given Nrf2's dual role in regulating both GPX4 expression and general antioxidant defenses, we assessed changes in oxidative stress markers in PTEN‐deficient APP/PS1 mice. Compared to Scramble controls, shPTEN treatment significantly upregulated key antioxidant proteins targeted by Nrf2, including HO‐1, NQO1, SOD1, SOD2 and NLRX1 (Figure 5A,B). Functional assessments confirmed these findings, as ROS levels, measured with DCFH‐DA probes, were significantly reduced (Figure 5C), while SOD activity levels were increased in shPTEN‐treated mice (Figure 5D). Furthermore, immunofluorescence analysis revealed reduced levels of 8‐hydroxy‐2'‐deoxyguanosine (8‐OHdG), a hallmark of oxidative DNA damage, in neurons from shPTEN‐treated mice (Figure 5E). Co‐staining with GFAP and Iba1 showed minimal 8‐OHdG signals in glial cells (Figure S10A,B). Altogether, these results demonstrate that PTEN knockdown attenuates oxidative stress by activating Nrf2‐dependent antioxidant pathways in APP/PS1 mouse brains.

**FIGURE 5:**
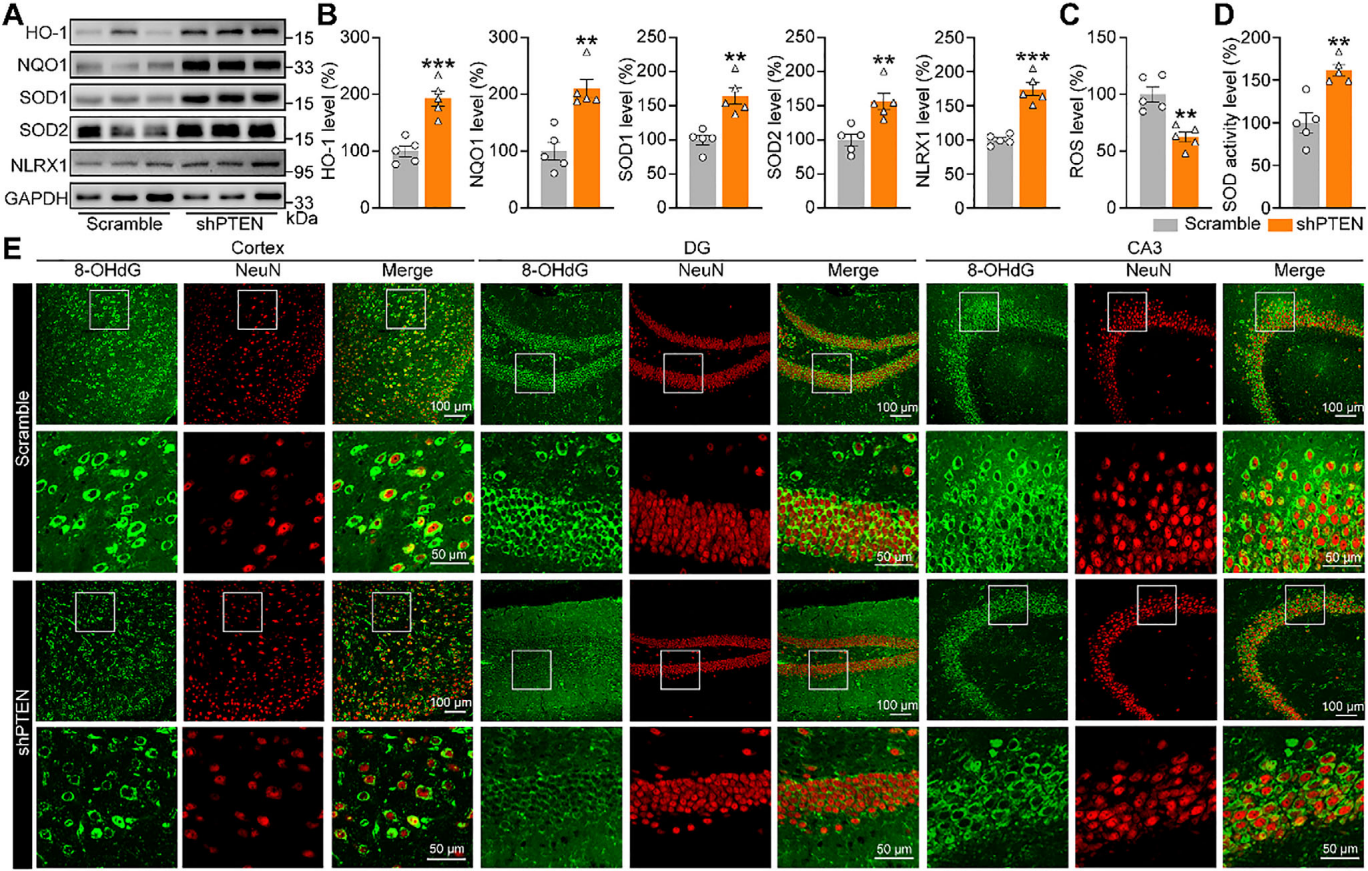
PTEN knockdown reduced oxidative stress in the brain of APP/PS1 mice. (A, B) Expression levels of HO‐1, NQO1, SOD1, SOD2 and NLRX1 were detected by Western blot. *n* = 5. (C, D) ROS levels and SOD activity in the mouse brain were separately determined using DCFH‐DA fluorescent probe and xanthine oxidase. *n* = 5. (E) Representative images showing the oxidative damage in the mouse brain using 8‐OHdG immunostaining. Scale bars, 100 µm and 50 µm. **p < 0.01, ***p < 0.001.

### 2.6 PTEN Knockdown Attenuated Oxidative Stress via AKT/GSK3β/Nrf2 and AKT/STAT3 Activation in N2a‐APPsw Cells

In order to determine whether PTEN knockdown up‐regulates GPX4 via the AKT/GSK3β/Nrf2 and AKT/STAT3 axis, we analyzed the activation of this pathway in siPTEN‐treated N2a‐APPsw cells. Western blot analysis showed that PTEN silencing substantially enhanced AKT and GSK3β phosphorylation (Figure 6A,B). PTEN knockdown increased Nrf2 protein levels and promoted its nuclear translocation, while co‐treatment with the Nrf2 inhibitor Nrf2‐IN1 reversed these effects (Figure 6C,D). PTEN knockdown also reduced the lipid peroxidation markers 4‐HNE and MDA and elevated the antioxidant proteins GPX4, SOD1, SOD2, HO‐1, and NQO1 (Figure 6E–H). In addition, siPTEN treatment upregulated both STAT3 and its phosphorylated form (p‐STAT3), whereas Stattic reversed the increase in p‐STAT3 and further enhanced total STAT3 expression (Figure 6I,J). Moreover, Stattic treatment decreased GPX4 expression while increasing the levels of 4‐HNE and MDA (Figure 6K,L).

**FIGURE 6:**
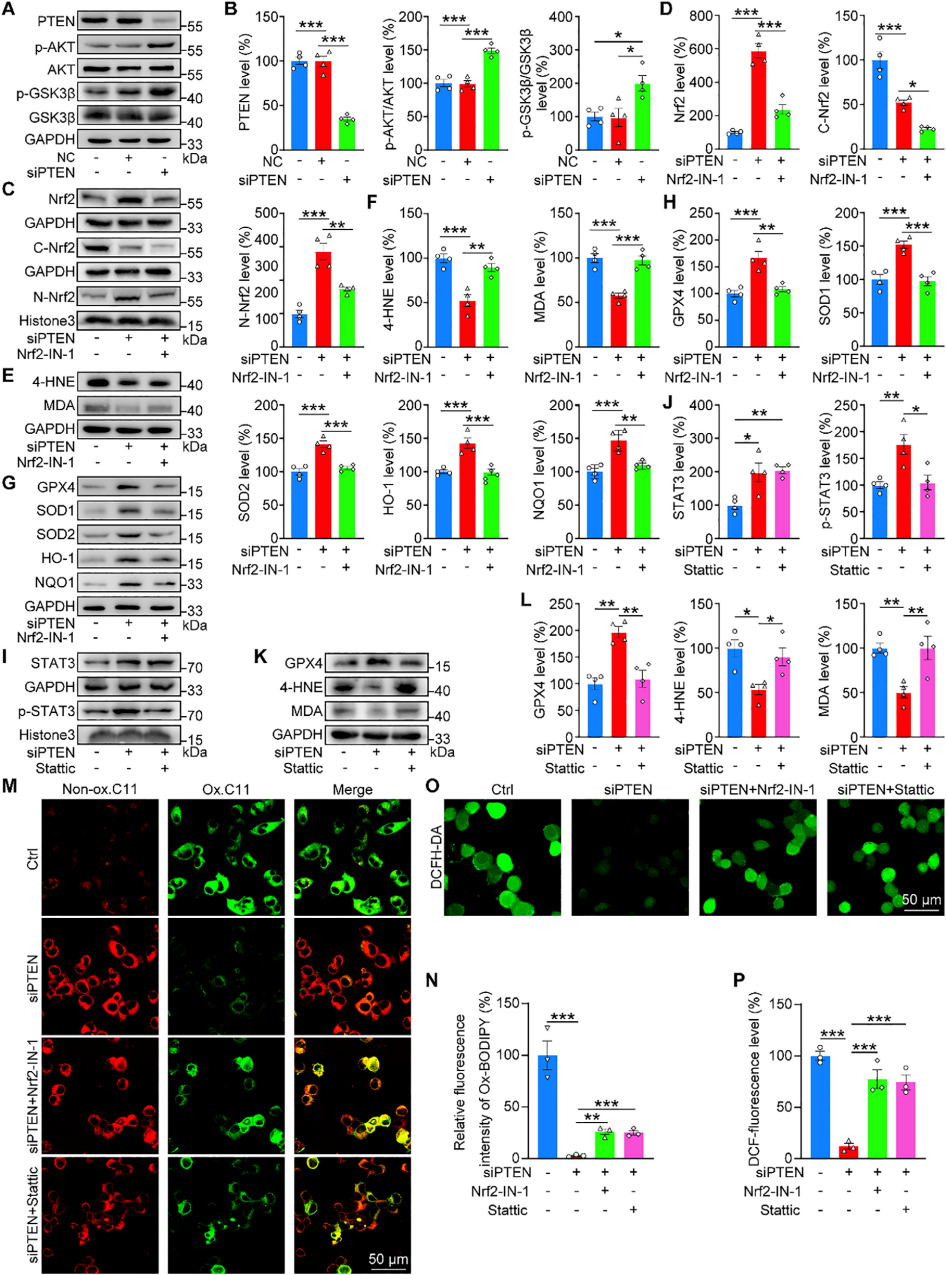
PTEN knockdown inhibited oxidative stress and lipid peroxidation via upregulating Nrf2 and p‐STAT3 expression in N2a‐APPsw cells. (A‐L) Protein expression of PTEN, p‐AKT, AKT, P‐GSK3β, GSK3β, Nrf2, C‐Nrf2, N‐Nrf2, 4‐HNE, MDA, GPX4, SOD1, SOD2, HO‐1, NQO1, STAT3 and p‐STAT3 were detected by Western blot. *n* = 4. (M‐P) Confocal microscopy visualized the changes in lipid peroxidation and ROS levels in N2a‐APPsw cells using C11‐BODIPY staining and DCFH‐DA staining, respectively. Scale bar, 50 µm. *n* = 3. *p < 0.05, **p < 0.01, ***p < 0.001.

Staining with the C11‐BODIPY 581/591 probe confirmed a reduction in lipid oxidation in siPTEN‐treated cells. This effect was reversed by Nrf2 and p‐STAT3 inhibition (Figure 6M,N). Similarly, DCFH‐DA assays showed a significant reduction in ROS following PTEN knockdown, which was restored by co‐treatment with Nrf2‐IN‐1 or Stattic (Figure 6O,P). These results confirm that PTEN reduction mitigated oxidative damage in N2a‐APPsw cells through AKT/GSK3β/Nrf2‐dependent and AKT/STAT3‐dependent mechanisms.

### 2.7 PTEN Silencing Attenuated RSL3‐Induced Ferroptosis via GSK3β/Nrf2/GPX4 and STAT3/GPX4 Signaling Pathways in N2a‐APPsw Cells

To further confirm that PTEN silencing attenuates ferroptosis rather than merely reducing ferroptosis‐associated oxidative damage, N2a‐APPsw cells were treated with the ferroptosis inducer RSL3 and the ferroptosis inhibitor Fer‐1 (Figure 7A–D). RSL3 significantly decreased GPX4 expression and increased 4‐HNE and MDA levels, whereas PTEN silencing markedly reversed these changes, accompanied by restored GSH levels and reduced lipid peroxidation. Fer‐1 similarly reduced MDA and 4‐HNE, while GPX4 expression remained largely unchanged, consistent with its role as a radical‐trapping antioxidant rather than a direct regulator of GPX4. To directly validate the role of GPX4, siGPX4 was applied. GPX4 knockdown increased MDA and 4‐HNE, whereas PTEN silencing showed opposite effects. Notably, siPTEN partially reversed siGPX4‐induced lipid peroxidation, indicating GPX4 as a key mediator of PTEN silencing (Figure S12A–D).

**FIGURE 7:**
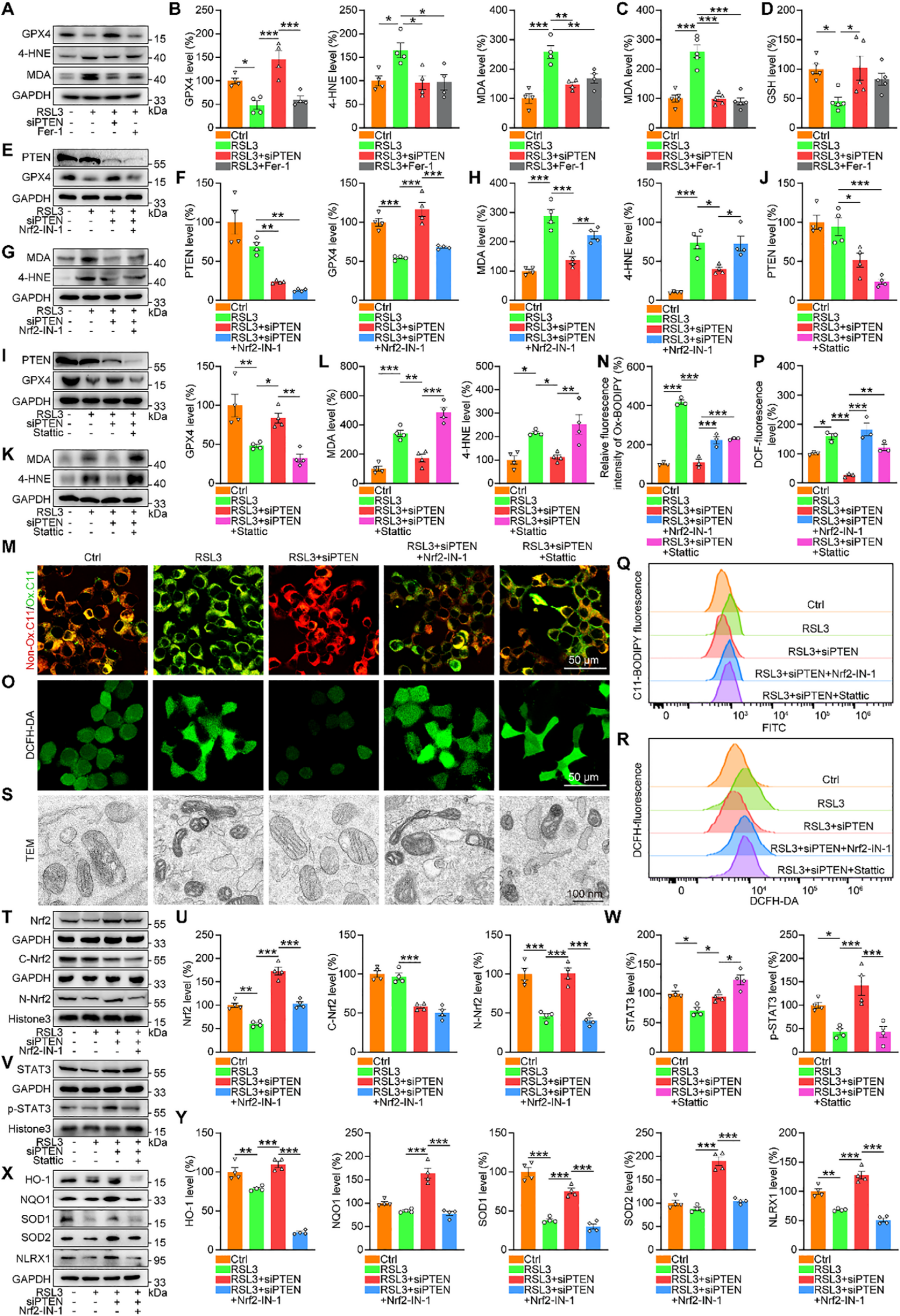
siPTEN inhibited ferroptosis by Nrf2/GPX4 signaling pathway in N2a‐APPsw cells. (A, B) Representative Western blot images showing protein expression of GPX4, 4‐HNE and MDA. *n* = 4. (C, D) MDA and GSH levels in N2a‐APPsw cells measured by assay kits. *n* = 5. (E‐L) Western blot analysis of PTEN, GPX4, MDA and 4‐HNE protein expression in N2a‐APPsw cells. *n* = 4. (M, N) Confocal microscopy visualized the alterations in lipid peroxidation in N2a‐APPsw cells after C11‐BODIPY probe staining. Scale bar, 50 µm. *n* = 3. (O, P) Representative images of DCFH‐DA staining in N2a‐APPsw cells. Scale bar, 50 µm. *n* = 3. (Q, R) Flow cytometric analysis showing the fluorescence intensity of C11‐BODIPY 581/591 staining and DCFH‐DA staining. (S) Representative TEM images of mitochondrial morphology. Scale bars, 100 nm. (T‐Y) Western blot assays to detect the expression of Nrf2, C‐Nrf2, N‐Nrf2, STAT3, p‐STAT3, HO‐1, NQO1, SOD1, SOD2 and NLRX1. *n* = 4. *p < 0.05, **p < 0.01, ***p < 0.001.

To delineate the underlying mechanism, we assessed the involvement of the Nrf2/GPX4 and p‐STAT3/GPX4 pathways. Immunoblotting detection showed that PTEN silencing rescued RSL3‐induced GPX4 suppression and reduced 4‐HNE and MDA, whereas these effects were significantly abrogated when combined with Nrf2‐IN‐1 or Stattic treatment (Figure 7E–L). Notably, combined inhibition of Nrf2 and STAT3 further intensified these effects compared with Nrf2‐IN‐1 alone, as evidenced by a more pronounced decrease in GPX4 and a greater increase in lipid peroxidation markers (Figure S14A–C). Furthermore, inhibition of AKT signaling with MK‐2206 reduced p‐GSK3β and nuclear p‐STAT3 levels, and suppressed Nrf2 expression and nuclear translocation. Consistently, MK‐2206 attenuated siPTEN‐induced GPX4 upregulation and restored lipid peroxidation, as indicated by increased MDA and 4‐HNE levels (Figure S11A–G). Collectively, these results indicate that PTEN knockdown alleviates ferroptosis in AD models at least in part through AKT‐dependent activation of the Nrf2/GPX4 and STAT3/GPX4 axes.

Functional assays further confirmed these observations. C11‐BODIPY 581/591 staining revealed that PTEN silencing significantly reduced RSL3‐triggered lipid peroxidation in an Nrf2‐dependent and p‐STAT3‐dependent manner (Figure 7M,N,Q; Figure S13A). This protective effect was consistently observed in parallel measurements of intracellular ROS levels using DCFH‐DA fluorescent probes (Figure 7O,P,R). Meanwhile, ultrastructural analysis demonstrated that PTEN knockdown ameliorated RSL3‐induced the ferroptosis‐like mitochondrial damage, which was similarly blocked by Nrf2‐IN‐1 and Stattic (Figure 7S; Figure S13B). Furthermore, PTEN knockdown restored RSL3‐suppressed antioxidant proteins (HO‐1, NQO1, SOD1, SOD2, NLRX1) through enhanced nuclear Nrf2 translocation and upregulation of p‐STAT3 (Figure 7T–Y).

These results clearly demonstrated that PTEN downregulation attenuated neuronal ferroptosis by activating the GSK3β/Nrf2/GPX4 and p‐STAT3/GPX4 pathways in N2a‐APPsw cells.

## 3 Discussion

### 3.1 Establishing PTEN as a Modulator of Ferroptosis in AD

Accumulating evidence has implicated PTEN dysregulation in synaptic dysfunction and cognitive decline in AD [17, 24], yet the molecular mechanisms linking PTEN to disease pathogenesis remain poorly understood. Here, we identify PTEN as a critical modulator of ferroptosis in AD. Aberrant PTEN upregulation disrupts AKT‐dependent signaling, leading to impaired activation of the GSK3β/Nrf2 and STAT3 pathways and consequent suppression of GPX4‐mediated antioxidant defense. This mechanism establishes PTEN as a key molecular link between AD‐associated signaling imbalance and ferroptosis‐driven neuronal degeneration.

In fact, ferroptosis is increasingly recognized as a key driver of AD progression and represents a promising therapeutic target [25, 26]. Deferoxamine, an iron chelator, was found to improve cognitive function in AD by suppressing ferroptosis [27]. Tetrahydroxy stilbene glycoside attenuates ferroptosis by enhancing GPX4 expression, thereby reducing Aβ production in the APP/PS1 mouse brain [28]. Furthermore, alpha‐lipoic acid significantly ameliorated cognitive impairment in P301S tau transgenic mice by inhibiting ferroptosis [29]. Interestingly, PTEN has been reported to promote ferroptosis by modulating iron homeostasis and lipid peroxide levels in septic myocardial injury [30]. Further studies have shown that PTEN deficiency in breast and prostate cancer cells activates the PI3K/AKT/mTOR pathway, resulting in SREBP activation, increased lipid synthesis and consequent suppression of ferroptosis [31]. These findings suggest that downregulation of PTEN in the brain may slow the progression of AD by inhibiting ferroptosis. Our study provides direct experimental evidence that PTEN downregulation effectively suppresses ferroptosis in APP/PS1 mice, as evidenced by reduced levels of lipid peroxidation products such as MDA and 4‐HNE.

### 3.2 Pten Knockdown Selectively Rescues GPX4‐dependent Antioxidant Defense

As a key regulator of ferroptosis, GPX4 prevents cell death by transforming lipid hydroperoxides into non‐toxic lipid alcohols [32, 33]. Previous work has shown that GPX4 expression is significantly reduced in AD brain, while genetic upregulation of GPX4 can rescue cognitive deficits by inhibiting neuronal ferroptosis [34, 35]. Notably, PTEN upregulation can drive GPX4 depletion, thereby accelerating the progression of ferroptosis [36]. Our findings demonstrate that PTEN reduction restores GPX4 levels, positioning PTEN downregulation as a potential therapeutic strategy for AD. Furthermore, ACSL4 serves as a critical executor of lipid peroxidation‐dependent ferroptosis [37], and previous work has shown that PTEN exacerbates ferroptosis via ACSL4 upregulation [38]. However, our study did not show a significant effect of PTEN on ACSL4 expression in AD models. Similarly, neither the existing literature nor our current data support PTEN‐mediated regulation of the ferroptosis suppressor FSP1. Systematic evaluation of iron transporters, including TFR1 and DMT1, further confirmed that PTEN downregulation does not alter their expression. In addition, direct measurement of brain ferrous iron levels revealed no significant differences between groups, further indicating that PTEN downregulation does not substantially affect iron accumulation under our experimental conditions. Notably, chronic metabolic disorders such as diabetes have been reported to influence brain iron homeostasis, with hyperglycemia associated with increased brain iron deposition and impaired neurovascular coupling potentially affecting cerebral iron regulation [39, 40]. Together, these results demonstrate that PTEN suppression inhibits ferroptosis in APP/PS1 mice predominantly through GPX4‐dependent mechanisms.

### 3.3 Pten Regulates GPX4 via Dual AKT‐driven Transcriptional Axes

To elucidate the underlying mechanisms underlying PTEN‐mediated GPX4 regulation, we performed RNA‐seq analysis on hippocampal tissues from PTEN‐knockdown APP/PS1 mice. The transcriptomic data showed significant enrichment of the PI3K/AKT pathway among differentially expressed genes, with Nrf2 and STAT3 identified as a key regulator through differential expression and KEGG pathway analyses. As a negative regulator of PI3K/AKT signaling [41, 42, 43], PTEN inhibition consequently activates AKT. This mechanistic relationship is supported by several lines of evidence. In a model of hepatic ischemia/reperfusion injury, PTEN downregulation enhances the efficacy of the ferroptosis inhibitor ME1 through activation of the AKT/PI3K pathway [19]. Similarly, PTEN silencing increases Nrf2 expression via AKT/PI3K activation in endplate chondrocytes [44]. Complementary studies show that AKT activation facilitates Nrf2 nuclear translocation [45]. Mechanistically, AKT activation induces phosphorylation and inactivation of GSK3β, a kinase that otherwise promotes Nrf2 nuclear export and proteasomal degradation [46]. Accordingly, the AKT/GSK3β/Nrf2 axis represents a central signaling cascade linking PTEN activity to redox homeostasis and ferroptosis regulation.

Recent studies have further demonstrated that Schisandrin B suppresses GSK3β activity, leading to Nrf2 activation and subsequent upregulation of GPX4, which ultimately attenuates neuronal ferroptosis [47]. As Nrf2 transcriptionally regulates GPX4 expression [48], its activation through the Nrf2/GPX4 axis effectively attenuates ferroptosis‐mediated neuroinflammation in the AD brain [49]. Collectively, these findings strongly suggest that PTEN modulates GPX4 expression primarily through the AKT/GSK3β/Nrf2 signaling cascade. Our results directly demonstrated that PTEN downregulation activated AKT, inhibited GSK3β activity, and consequently increased Nrf2 expression and promoted its nuclear accumulation in APP/PS1 mice. In addition, STAT3 plays a crucial role in regulating antioxidant defense and cell survival. As a transcriptional activator, p‐STAT3 can protect cells from ferroptotic damage by promoting GPX4 expression. Ye C et al. reported that delivery of a STAT3 inhibitor elevated p‐STAT3 levels, thereby upregulating GPX4 and inducing ferroptosis, ultimately exerting an anti‐colorectal cancer effect [50]. Importantly, phosphorylated AKT has been shown to promote STAT3 activation [51]. Based on this evidence, we speculate that the AKT/STAT3/GPX4 signaling axis may also play a critical role in suppressing ferroptosis in the AD model. Our results demonstrated that PTEN knockdown upregulated p‐STAT3 expression, thereby enhancing GPX4 levels and inhibiting neuronal ferroptosis in APP/PS1 mice. These findings suggest that the AKT/STAT3/GPX4 axis represents an additional pathway contributing to the anti‐ferroptotic effects of PTEN inhibition. Taken together, these findings suggest that PTEN inhibition exerts anti‐ferroptotic effects in AD by activating the AKT/GSK3β/Nrf2/GPX4 and AKT/STAT3/GPX4 signaling pathways.

### 3.4 Broader Implications for Oxidative Stress Modulation in AD

Notably, in addition to regulating GPX4, Nrf2 serves as a master regulator of cellular oxidative stress responses. Upon nuclear import, Nrf2 binds to antioxidant response elements (ARE) to transcriptionally activate a number of antioxidant genes [52]. The link between PTEN and oxidative stress regulation is supported by several lines of evidence. PTEN inhibition activates the PI3K/AKT pathway, which has a potent antioxidant effect [53], while AKT activation resulting from PTEN suppression further inhibits critical mediators of oxidative stress, thereby attenuating both AD‐related pathology and cognitive decline [54]. Moreover, PTEN downregulation directly enhances Nrf2 expression [55]. Fourth, PTEN deficiency attenuates ferroptosis by reducing oxidative damage [56]. Taken together, these previous findings establish PTEN as a key regulator of oxidative stress. Our current results not only confirm these existing observations, but also provide additional mechanistic insights, demonstrating that PTEN downregulation significantly alleviates oxidative stress via coordinated mechanisms. Specifically, it reduces ROS generation while increasing the expression of key antioxidant enzymes, including HO‐1, NQO1, SOD1, SOD2, and NLRX1, and significantly reduces 8‐OHdG levels. Moreover, PTEN knockdown elevated intracellular GSH levels, further supporting enhanced antioxidant capacity; however, the precise mechanism underlying GSH elevation was not systematically investigated in this study and therefore remains to be clarified. Our findings provide compelling evidence that PTEN suppression is an effective approach to mitigate oxidative stress in AD pathogenesis.

## 4 Conclusions

In conclusion, our results demonstrate that PTEN knockdown significantly improves cognitive function in APP/PS1 mice. Mechanistic studies reveal that PTEN downregulation suppresses ferroptosis by activating of the AKT/GSK3β/Nrf2/GPX4 and AKT/STAT3/GPX4 pathways. Furthermore, PTEN downregulation appears to inhibit oxidative stress by promoting Nrf2 translocation to the nucleus (Figure 8). Therefore, these novel findings position PTEN as a promising therapeutic target for AD intervention.

**FIGURE 8:**
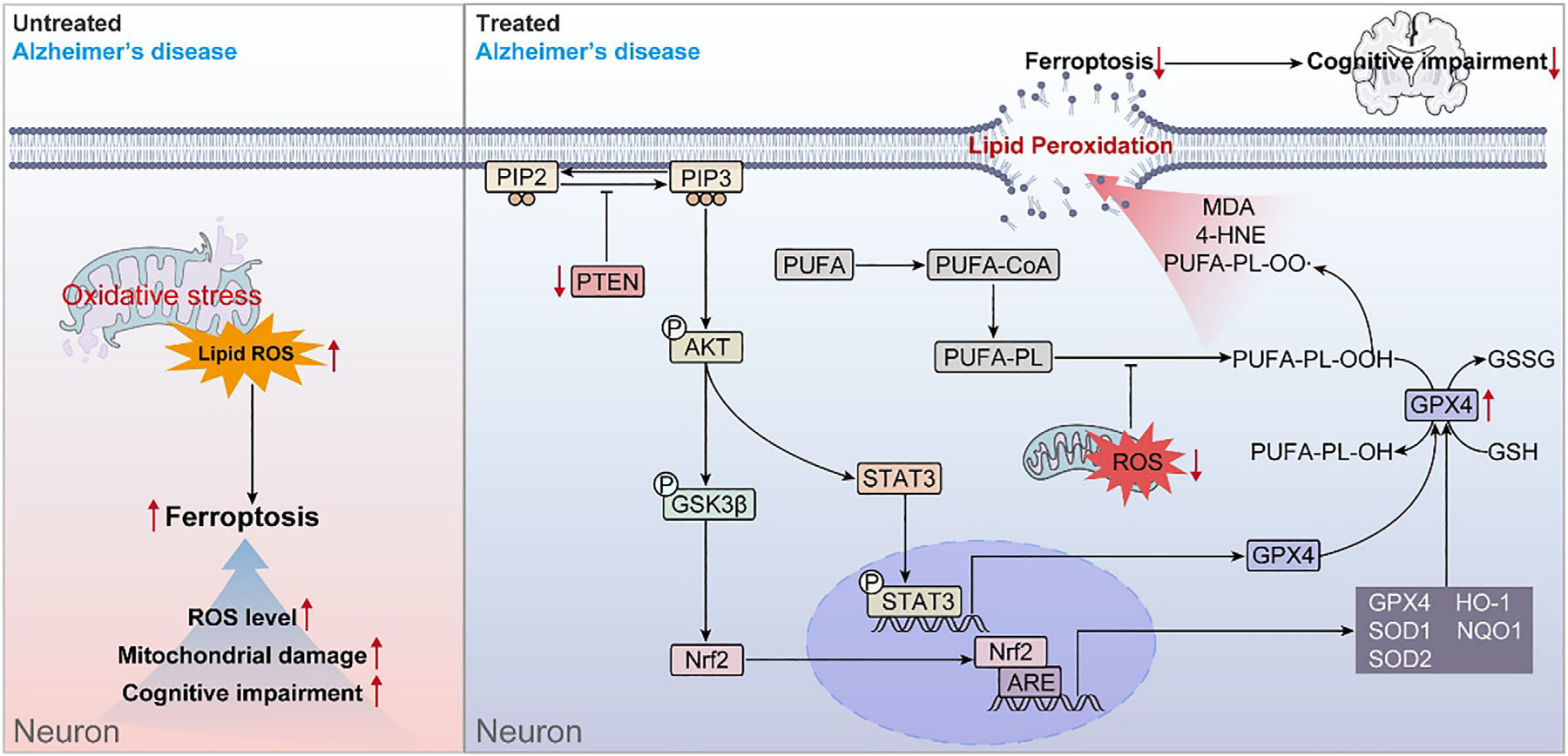
Schematic showing that PTEN knockdown inhibited neuronal ferroptosis in APP/PS1 transgenic mice. PTEN knockdown promoted AKT phosphorylation, which in turn enhanced Nrf2 nuclear translocation and p‐STAT3 nuclear expression. This led to upregulation of GPX4, along with increased expressions of HO‐1, SOD1, SOD2, and NQO1, reducing PL‐OH content and ROS levels. Collectively, these changes synergistically inhibited ferroptosis and ultimately ameliorated cognitive impairment in APP/PS1 transgenic mice.

## 5 Experimental Section

### 5.1 Databases and Bioinformatic Analysis

AD‐related genes were retrieved from the GeneCards database (https://www.genecards.org/) using the keyword“Alzheimer's disease”. Genes were ranked according to the GeneCards Inferred Functionality Score (GIFtS), and the top 200 genes were selected for subsequent analyses. Ferroptosis‐related genes were obtained from the FerrDb database (http://www.zhounan.org/ferrdb/current/). The overlap between AD‐related and ferroptosis‐related genes was evaluated using a one‐sided Fisher's exact test (hypergeometric test) based on all human protein‐coding genes as the background universe. The GSE122063 dataset, comprising temporal cortex samples from AD patients and controls, was downloaded from GEO (https://www.ncbi.nlm.nih.gov/geo/). Differential expression analysis was conducted using the *limma* package in R, and the results were visualized with the *pheatmap* package.

### 5.2 Human Brain Samples

Human brain tissues were fixed in formaldehyde and sectioned at 40 µm thickness. Samples included a control (P535‐00) and a late‐stage AD case (T4339; an 88‐year‐old female). The experiments were performed according to guidelines of the New York Brain Bank at Columbia University (Alzheimer's Disease Research Center, Taub Institute) and of the approval of Lund University (Dnr 286–2014). All subjects gave written informed consent in accordance with the Declaration of Helsinki.

### 5.3 Transgenic Mice and Approval

Adult male APP/PS1 transgenic mice were obtained from The Jackson Laboratory (Bar Harbor, ME, USA). All mice were housed under standard conditions (22‐25°C, 12 h light/dark cycle). The experiments adhered to the “Laboratory Animals‐Guideline of welfare and ethics, The Ethics Committee for Medical Laboratory Animals of China Medical University”. Protocol approval was obtained from the Laboratory Animal Welfare and Ethics Committee of the China Medical University (No. CMU2021440).

### 5.4 Cell Culture

N2a‐APPsw cells, provided by Professor Huaxi Xu in Xiamen University, were cultured in a 37°C incubator with 5% CO_2_. PTEN‐targeting siRNA plasmids were constructed by Shanghai Sunbio Medical Biotechnology. For transfection, the siPTEN (2.5 µg/mL) was introduced into N2a‐APPsw cells using SuperKine Lipo3.0 Efficient Transfection Reagent (BMU111‐EN, Abbkine). In one set of in vitro experiments, cells were transfected with siPTEN and incubated in 10% FBS medium for 24 h, followed by 6 h of serum starvation. Then, either Nrf2‐IN‐1 (1 µM) (HY‐101025, MedChemExpress), Stattic (1 µM) (HY‐13818, MedChemExpress) or MK‐2206 (1 µM) (HY‐108232, MedChemExpress) was added, and cells were cultured for another 24 h. In another experiment, after siPTEN transfection, RSL3 (5 µM) (HY‐100218A, MedChemExpress) or Ferrostatin‐1 (1 µM) (HY‐100579, MedChemExpress) was added and cultured for 4 h, followed by the addition of Nrf2‐IN‐1, Stattic or MK‐2206, and cells were cultured for an additional 24 h.

### 5.5 Virus Injection

APP/PS1 transgenic mice were bilaterally injected in hippocampus with either AVV‐shPTEN or AAV‐Scramble (1×10^12^ vg/mL, 2 µL per side). Briefly, mice were anesthetized and positioned in a stereotaxic frame. Injections were performed at coordinates 2.5 mm to the lateral, 2.0 mm to the posterior, and 2.0 mm below bregma. The needle was retained for 5 min after injection to facilitate viral diffusion. After surgery, incisions were sealed, and mice received antibiotics and supportive care.

### 5.6 Morris Water Maze

Spatial learning and memory were evaluated using the Morris water maze. The apparatus consisted of a circular pool (90 cm in diameter and 40 cm in depth) containing an escape platform (8 cm in diameter), with water maintained at 18–23°C. Mice underwent 2 days of visible platform training, followed by 5 days of hidden platform trials. On day 8, a probe test was performed in which the platform was removed to assess spatial memory retention. All trials were recorded using the SMART 3.0 computer tracking program.

### 5.7 Nest Building Tests

The nesting experiment was conducted to assess the nesting behavior of mice. Mice were housed individually in cages, and eight 5 × 5 cm pieces of paper were placed in the same position in each cage. Photographs were taken at fixed intervals over 7 days. Behavioral changes were categorized as follows: 1‐no torn paper and scattered pieces; 2‐intact paper pieces clustered together; 3‐partially torn paper pieces; 4‐extensively torn and clustered pieces; 5‐paper pieces built into a complete nest.

### 5.8 Western Blot

Protease inhibitor (HY‐K0010, Med Chem Express) and phosphatase inhibitor (HY‐K0021, Med Chem Express) were added to the RIPA lysate (P0012B, Beyotime). Total protein levels were assessed using the BCA protein assay kit (T9300A, Takara). Protein samples (30 µg) were subjected to 8%‐12% SDS‐PAGE and transferred onto PVDF membranes for subsequent analysis (0.45 µm pore size).

After transfer, membranes were blocked in 5% milk for 30 min at room temperature. Primary antibodies were incubated overnight at 4°C (see Supplementary Table for antibody information), followed by incubation with HRP‐conjugated secondary antibodies for 1 h at room temperature. Images were captured using a gel imaging system (5200, Tanon).

### 5.9 Immunofluorescence Staining

Human brain slices (40 µm) or mouse brain slices (25 µm) were antigen repaired using sodium citrate solution at 60°C for 30 min. Sections were then blocked with goat serum for 1 h at room temperature and incubated overnight at 4°C with primary antibodies (see Table S1 for antibody information). Slices were incubated with fluorescent secondary antibodies for 2 h at room temperature and counterstained with DAPI for 5 min to visualize nuclei. Finally, sections were mounted with an anti‐fade reagent, and images were acquired by a confocal laser scanning microscope (A1, Nikon).

### 5.10 Immunohistochemical Staining

Mouse brain tissues were sequentially dehydrated through graded ethanol solutions and cleared with xylene, followed by paraffin infiltration, embedding, and sectioning at 5 µm. Sections underwent antigen retrieval in sodium citrate solution, and staining was subsequently performed according to the kit instructions (SV0001, BOSTER) or (SV0002, BOSTER). Details of the Primary antibodies are presented in Table S1.

### 5.11 Flow Cytometric Analysis

After the indicated treatment, the medium was removed from the N2a‐APPsw cells and DMEM containing DCFH‐DA or BODIPY 581/591C11 was added. Following incubation, cells were digested, washed with PBS, pelleted by centrifugation at 1000 rpm for 3 min, and subsequently resuspended in PBS. Finally, cellular fluorescence was measured using flow cytometry (FACSCelesta, BD).

### 5.12 Measurement of ROS

ROS generation was evaluated using the Reactive Oxygen Species Assay Kit (S0022M, Beyotime). Mouse hippocampal tissues were enzymatically dissociated, and the isolated cells were treated with DCFH‐DA at 37°C for 1 h. Fluorescence intensity was detected at 488 nm. For N2a‐APPsw cells, after DCFH‐DA incubation, ROS fluorescence was visualized by confocal microscopy and quantified by flow cytometry.

### 5.13 Measurement of GSH

GSH levels in hippocampal tissue were measured using the GSH Assay Kit (MAK517, Sigma‐Aldrich). Tissues were homogenized in MES buffer containing EDTA and centrifuged at 10,000 g for 15 min at 4°C. The supernatant was mixed with assay reagents. Absorbance was measured at 412 nm.

### 5.14 Measurement of Total SOD Activity

Total SOD activity in samples was assessed using the Total SOD Activity Assay Kit (MAK528‐1KT, Sigma‐Aldrich). Samples were homogenized in cold lysis buffer and supernatants were incubated with working reagents and xanthine oxidase. Absorbance was measured at 440 nm.

### 5.15 Extraction of Nuclear Proteins

Nuclear and cytoplasmic proteins were extracted using the NE‐PER kit (78833, Thermo Fisher Scientific). Following the manufacturer's protocol, N2a‐APPsw cells and 50 mg of hippocampal tissue were processed, and the resulting nuclear extracts were kept at ‐80°C.

### 5.16 Transmission Electron Microscopy

Mitochondrial ultrastructure was examined by TEM. Fresh tissue was fixed in 2.5% glutaraldehyde at 4°C overnight, followed by post‐fixation in 1% osmium acid for 2 h at room temperature. After graded dehydration in ethanol and acetone, samples were sectioned into ultrathin slices (60‐80 nm) and sequentially stained with 2% uranyl acetate and 2.6% lead citrate. Images were taken using a TEM (HT7800/HT7700, Hitachi).

### 5.17 Lipid Peroxidation Assay

Lipid peroxidation was evaluated using the BODIPY 581/591C11 assay kit (D3861, Invitrogen). N2a‐APPsw cells were incubated with the probe in culture medium at 37°C for 1 h. Fluorescence was visualized by confocal microscopy or quantified by flow cytometry. The red‐to‐green fluorescence ratio was used to indicate lipid peroxidation levels.

### 5.18 Statistical Analysis

Data are presented in this paper as mean ± standard errors (SEM) from 3 replicate experiments. Differences between two groups were assessed using Student's *t*‐test, while one‐way ANOVA was employed for comparisons among multiple groups. Data analysis was performed using Image J and GraphPad Prism 9. *P* < 0.05 was considered statistically significant.

## Author Contributions

D.W.W. was responsible for creating the figures and drafting the manuscript. M.M.L. performed the experiments and conducted the bioinformatics analysis. J.Y.L. and Y.C.Z. supplied study‐specific materials and relevant details. W.L. and X.Y. conceptualized and supervised the study secured the funding for the project. All authors reviewed the manuscript and provided feedback to enhance its quality.

## Funding

This work was supported by the National Natural Sciences Foundation of China (81901116). Department of Science and Technology of Liaoning Province, 2024JH6/100800008. Department of Education of Liaoning Province, LJKMZ20221207.

## Ethical Statement

This study was approved by the Ethics Committee for Experimental Animal Welfare of China Medical University (No. CMU2021440).

## Conflicts of Interest

The authors declare no conflicts of interest.

## Supporting information




**Supporting File**: advs76989‐sup‐0001‐SuppMat.docx.

## Data Availability

The data that support the findings of this study are available from the corresponding author upon reasonable request.
