# Supplementary material for: Targeting Hippocampal PTEN Suppresses Ferroptosis and Rescues Cognitive Decline in Alzheimer's Disease via Dual AKT/GSK3β/Nrf2 and AKT/STAT3 Axes
Source: Adv Sci (Weinh). 2026 Aug 3:e76989. Online ahead of print. doi: 10.1002/advs.76989 (PMC13430936; doi:10.1002/advs.76989)
Supplement: Supplementary file 1 — Supporting File: advs76989‐sup‐0001‐SuppMat.docx. [file ADVS-9999-e76989-s001.docx]

Supporting Information

**Targeting Hippocampal PTEN Suppresses Ferroptosis and Rescues Cognitive Decline in Alzheimer's Disease via Dual AKT/GSK3β/Nrf2 and AKT/STAT3 Axes**

Da-Wei Wang^†^, Meng-Meng Liu^†^, Yu-Chen Zhao, Jia-Yi Li, Wen Li*, Xin Yu*

(^†^ These authors contributed equally to this work)

(* Corresponding authors)

Xin Yu, Da-Wei Wang

Key Laboratory of Medical Cell Biology of Ministry of Education, Key Laboratory of Major Chronic Diseases of Nervous System of Liaoning Province, Health Sciences Institute of China Medical University, Shenyang, China.

E-mail: yuxin@cmu.edu.cn (Corresponding author), [77776583@cmu.edu.cn](mailto:77776583@cmu.edu.cn).

Wen Li, Jia-Yi Li

Laboratory of Research in Parkinson's Disease and Related Disorders, Liaoning Provincial Key Laboratory of Major Neurological Diseases, Health Sciences Institute, China Medical.

E-mail: wli87@cmu.edu.cn (Corresponding author), lijiayi@cmu.edu.cn.

Meng-Meng Liu

Key Laboratory of Medical Cell Biology of Ministry of Education, Key Laboratory of Major Chronic Diseases of Nervous System of Liaoning Province, Health Sciences Institute of China Medical University, Shenyang, China; Department of Neurology, Second Affiliated Hospital, Army Medical University (Third Military Medical University).

E-mail: mmliu@cmu.edu.cn.

Yu-Chen Zhao

Department of Physical Education, Institute of China Medical University, Shenyang, China.

E-mail: yczhao@cmu.edu.cn.

**Table 1.** List of antibodies used for Western blot, immunofluorescent, and immunohistochemistry.

| **Antibody** | **WB** | **IF** | **IHC** | **Source** | **#Catalog** |
| --- | --- | --- | --- | --- | --- |
| Anti-Aβ | NA | 1:400 | NA | Santa Cruz | Sc-28365 |
| Anti-ACSL4 | 1:1000 | NA | NA | Sigma-Aldrich | SAB2701949 |
| Anti-AKT | 1:1000 | NA | NA | Cell Signaling Technology | 2920S |
| Anti-DMT1 | 1:1000 | NA | NA | Proteintech Group, Inc | 20507-1-AP |
| Anti-FSP1 | 1:1000 | NA | NA | Abcam | ab124805 |
| Anti-GFAP | NA | 1:200 | NA | Cell Signaling Technology | 3670 |
| Anti-GFAP | NA | 1:200 | NA | Cell Signaling Technology | HPA056030 |
| Anti-GPX4 | 1:1000 | NA | 1:100 | Abcam | ab125066 |
| Anti-GPX4 | NA | 1:100 | NA | Sigma-Aldrich - | SAB4300725 |
| Anti-GAPDH | 1:1000 | NA | NA | Abcam | ab8245 |
| Anti-GSK3β | 1:1000 | NA | NA | Immunoway | YM8575 |
| Anti- Histone | 1:1000 | NA | NA | Cell Signaling Technology | 4499S |
| Anti-HO-1 | 1:1000 | NA | NA | Cell Signaling Technology | 70081S |
| Anti-Iba1 | NA | 1:200 | NA | FUJIFILM Wako Pure Chemical Corporation | 019-19741 |
| Anti-Iba1 | NA | 1:400 | NA | Abcam | AB283319 |
| Anti-IDE | 1:1000 | NA | NA | Thermo Fisher Scientific | PA5-29350 |
| Anti-Keap1 | 1:1000 | NA | NA | Affinity Biosciences | AF5266 |
| Anti-LRP1 | 1:50000 | NA | NA | Abcam | ab92544 |
| Anti-MDA | 1:1000 | NA | NA | Abcam | Ab27642 |
| Anti-NeuN | NA | 1:400 | NA | Invitrogen | 702207 |
| Anti-NeuN | NA | 1:400 | NA | Invitrogen | MA5-33103 |
| Anti-NQO1 | 1:1000 | NA | NA | Thermo Fisher Scientific | MA1-16672 |
| Anti-NLRX1 | 1:1000 | NA | NA | Cell Signaling Technology | 13829S |
| Anti-Nrf2 | 1:1000 | NA | 1:100 | Thermo Fisher Scientific | PA5-27882 |
| Anti-p-AKT | 1:1000 | NA | NA | Cell Signaling Technology | 4060S |
| Anti-p-GSK3β | 1:1000 | NA | NA | Immunoway | YM8041 |
| Anti-PI3K | 1:1000 | NA | NA | Thermo Fisher Scientific | MA1-74183 |
| Anti-p-PI3K | 1:1000 | NA | NA | Thermo Fisher Scientific | PA5-17387 |
| Anti- PSD95 | NA | 1:100 | NA | Servicebio | GB11277 |
| Anti-PTEN | 1:1000 | 1:100 | 1:100 | Proteintech | 22034-1AP |
| Anti-p-STAT3 | 1:1000 | NA | 1:100 | Immunoway | YM8551 |
| Anti-SOD1 | 1:1000 | NA | NA | Thermo Fisher Scientific | MA1-105 |
| Anti-SOD2 | 1:1000 | NA | NA | Thermo Fisher Scientific | MA1-106 |
| Anti-STAT3 | 1:1000 | NA | NA | Immunoway | YM8325 |
| Anti-Tau396 | NA | 1:200 | NA | ImmunoWay | YP0625 |
| Anti-Tau231 | NA | 1:200 | NA | Sigma-Aldrich | SAB4504563 |
| Anti- Synaptophysin | NA | 1:400 | NA | Invitrogen | 710532 |
| Anti-TFR1 | 1:1000 | NA | NA | Abcam | ab214039 |
| Anti-4-HNE | 1:1000 | NA | NA | Biosynthesis Biotechnology | BS-6313R |
| Anti-β-actin | 1:1000 | NA | NA | Abcam | Ab8226 |
| Anti-8-OHdG | NA | 1:200 | NA | Abcam | ab183393 |
| Anti-8-OHdG | NA | 1:400 | NA | Santa Cruz | sc-66036 |
| Anti-NeuN | NA | 1:100 | NA | Abcam | ab177487 |
| Goat anti-Rabbit IgG (H+L) Cross-Adsorbed Secondary Antibody, Alexa Fluor™ 488 | NA | 1:400 | NA | Invitrogen | A11008 |
| Goat anti-Mouse IgG (H+L) Cross-Adsorbed Secondary Antibody, Alexa Fluor™ 488 | NA | 1:400 | NA | Invitrogen | A11001 |
| Goat anti-Mouse IgG (H+L) Cross-Adsorbed Secondary Antibody, Alexa Fluor™ 594 | NA | 1:400 | NA | Invitrogen | A11005 |
| Goat anti-Rabbit IgG (H+L) Cross-Adsorbed Secondary Antibody, Alexa Fluor™ 594 | NA | 1:400 | NA | Invitrogen | A11012 |

**Supplementary Figures.**


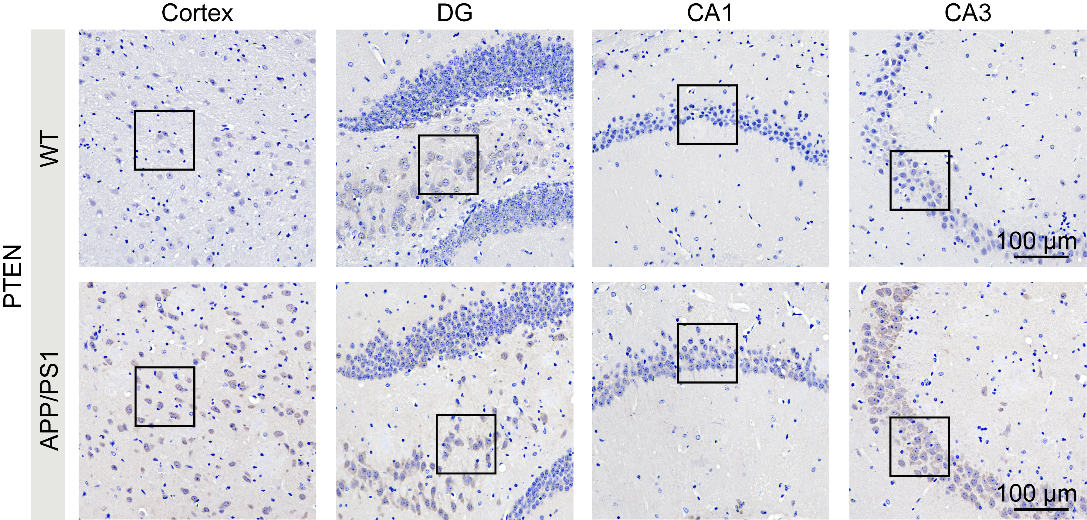


**Figure S1.** Representative images of anti-PTEN immunohistochemical staining in the cortex, DG, CA1, and CA3 regions of WT and APP/PS1 mouse brains. The black selection indicates the enlarged area and is shown in Figure 1E. Scale bar, 100 μm.

**
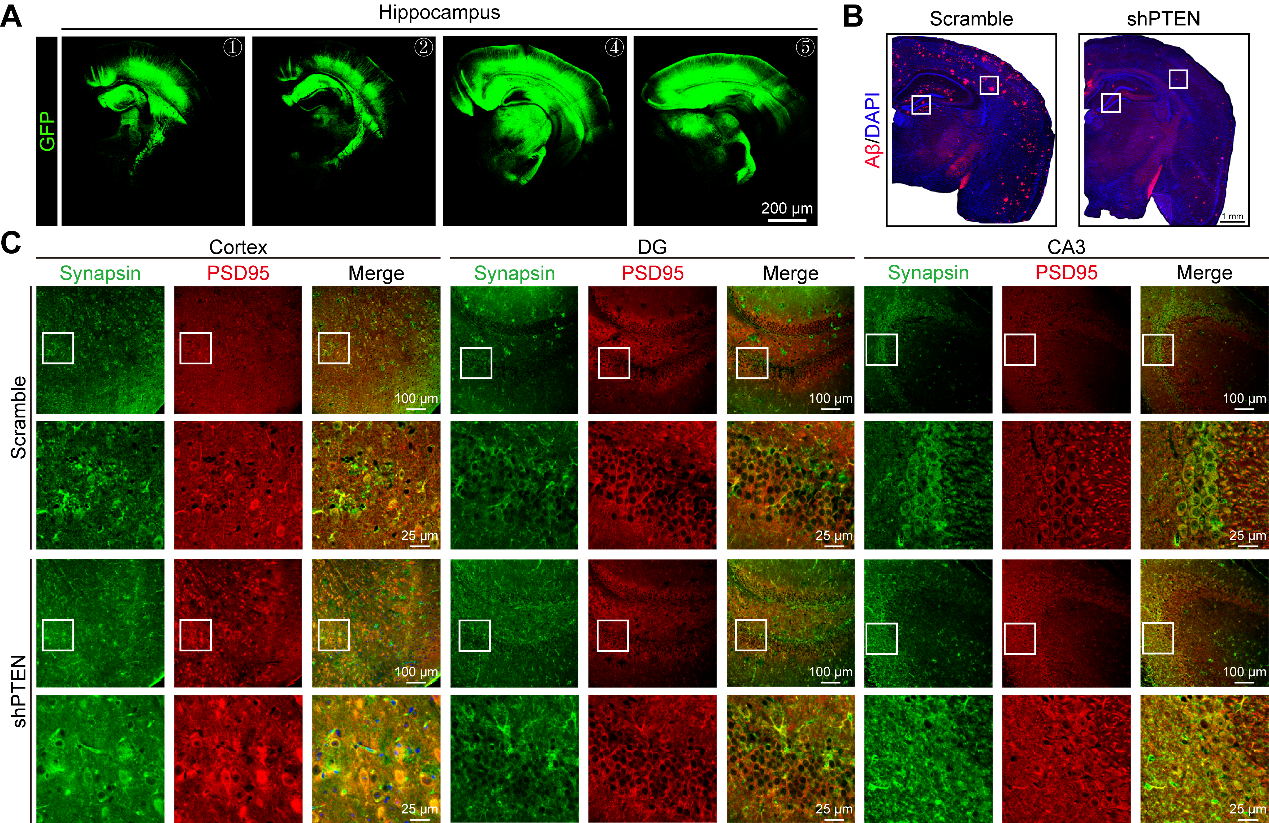
**

**Figure S2.** A) Representative fluorescence distribution in different hippocampal regions of APP/PS1 mice 5 weeks after hippocampal injection of GFP-labeled AAV-shPTEN. The selected region from layer③is presented in Figure 2C. Scale bar, 200 μm. B) Representative immunofluorescence staining of Aβ plaques in the cortex and hippocampus of APP/PS1 mouse brains. The white boxed area indicates the enlarged region shown in Figure 2K. Scale bar, 1 mm. C) Representative immunofluorescence images of Synapsin and PSD95 in different brain regions of mice. Scale bars, 100 μm and 25 μm.

**
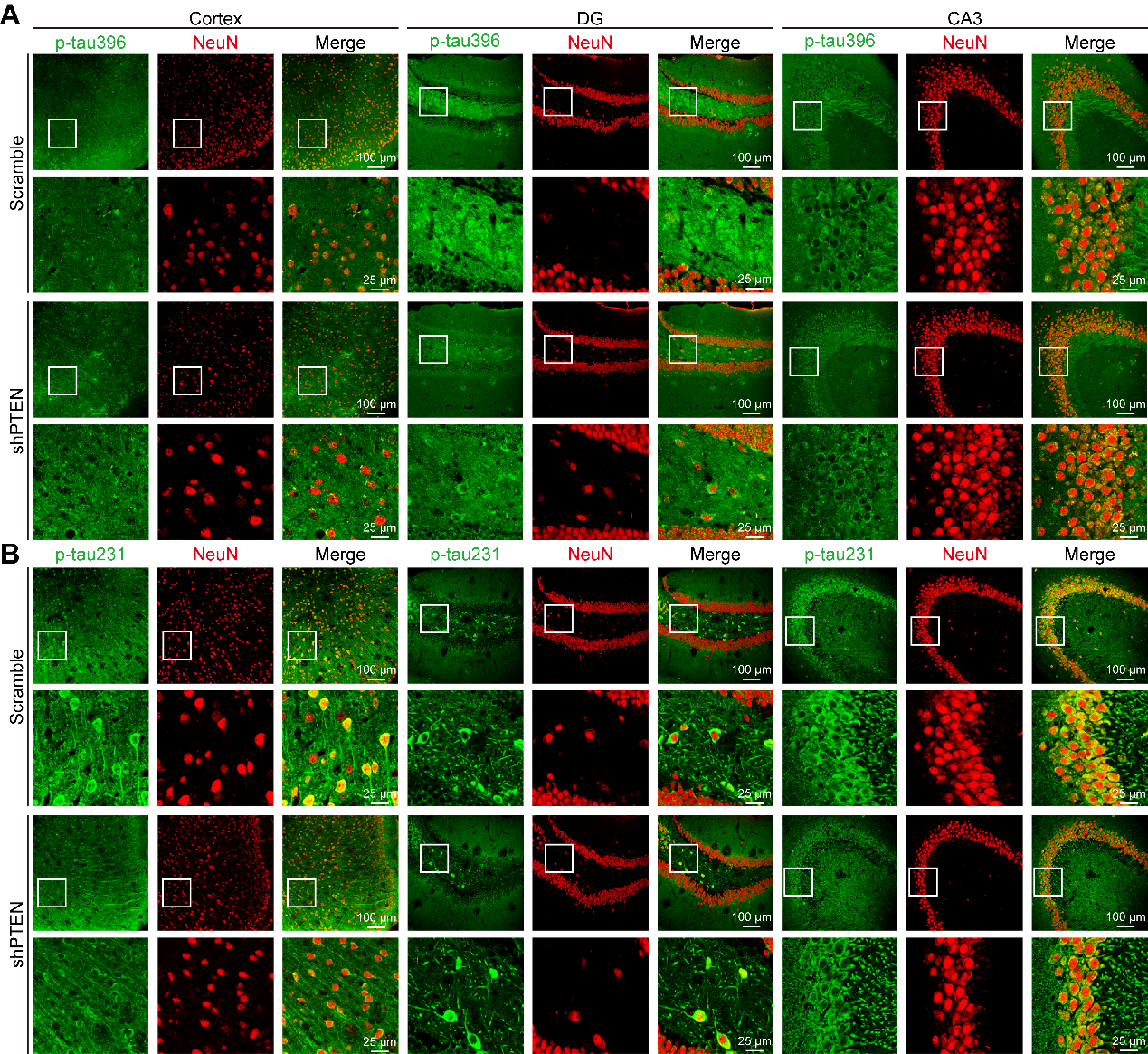
**

**Figure S3.** A, B) Representative immunofluorescence images of p-Tau396/NeuN and p-Tau231/NeuN staining in different brain regions of APP/PS1 transgenic mice. Scale bars, 100 μm and 25 μm.

**
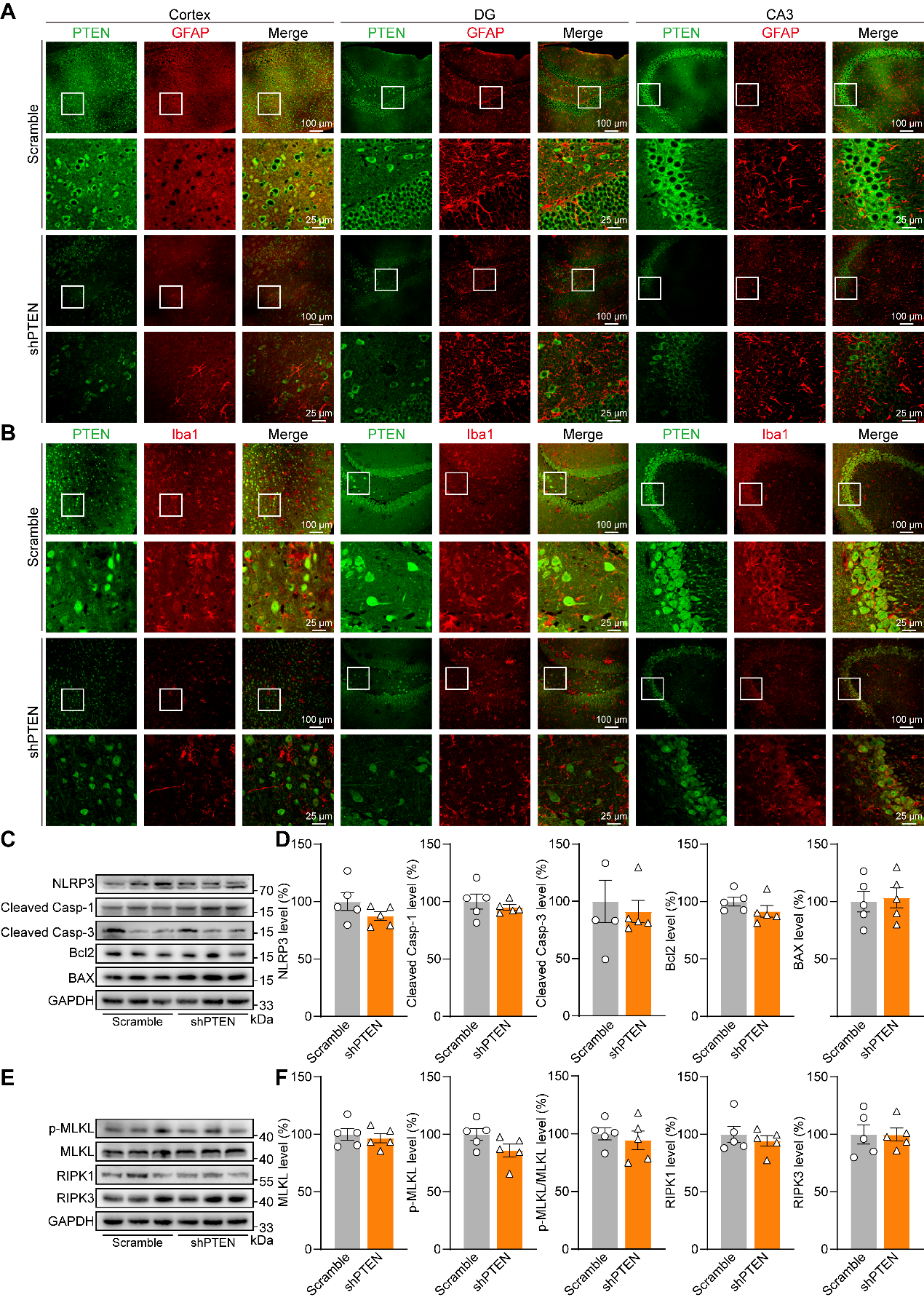
**

**Figure S4.** A, B) Representative immunofluorescence images of PTEN/GFAP and PTEN/Iba1 staining in different brain regions of APP/PS1 transgenic mice. Scale bars, 100 μm and 25 μm. C-F) Western blot analysis of NLRP3, Cleaved Caspase-1, Cleaved Caspase-3, Bcl2, BAX, p-MLKL, MLKL, RIPK1 and RIPK3 expression in mouse tissues. *n* = 5.


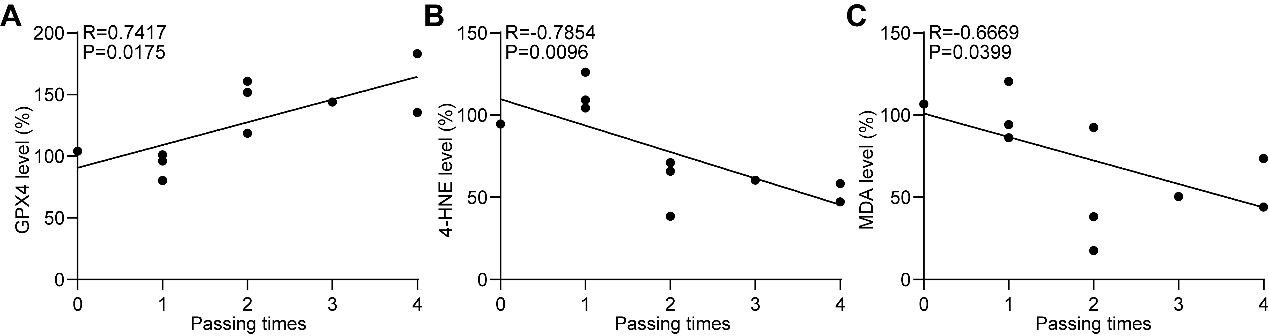


**Figure S5.** A-C) Correlation between ferroptosis markers and cognitive performance. Spearman correlation analysis between MWM day 8 platform crossing times and GPX4 (R = 0.7417, *P* = 0.0175), 4-HNE (R = 0.7854, *P* = 0.0096), and MDA (R = 0.6669, *P* = 0.0399) levels. *n* = 10.


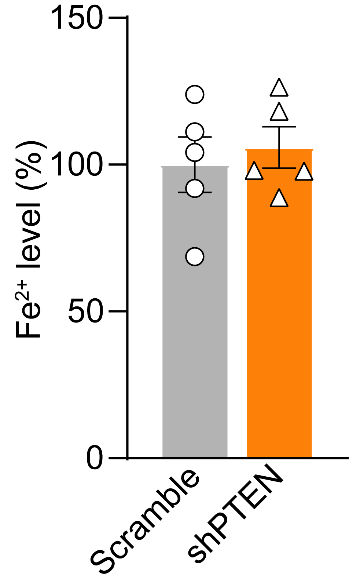


**Figure S6.** Quantification of ferrous iron (Fe^2+^) levels in the brain tissues of APP/PS1 transgenic mice. *n* = 5.

**
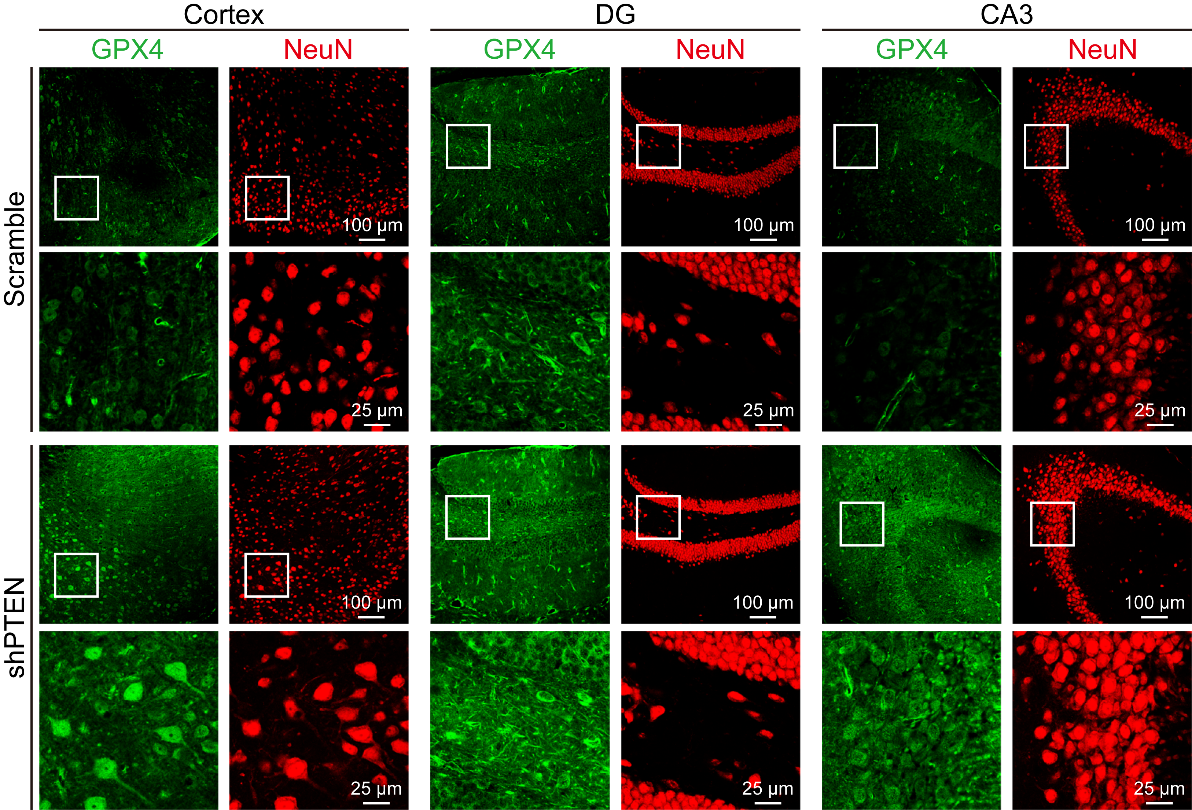
**

**Figure S7.** Representative immunofluorescence images of GPX4/NeuN co-staining showing GPX4 distribution in the cortex, DG, and CA3 regions of the hippocampus in APP/PS1 mice. Merged images are shown in Figure 3L. Scale bars, 100 μm and 25 μm.


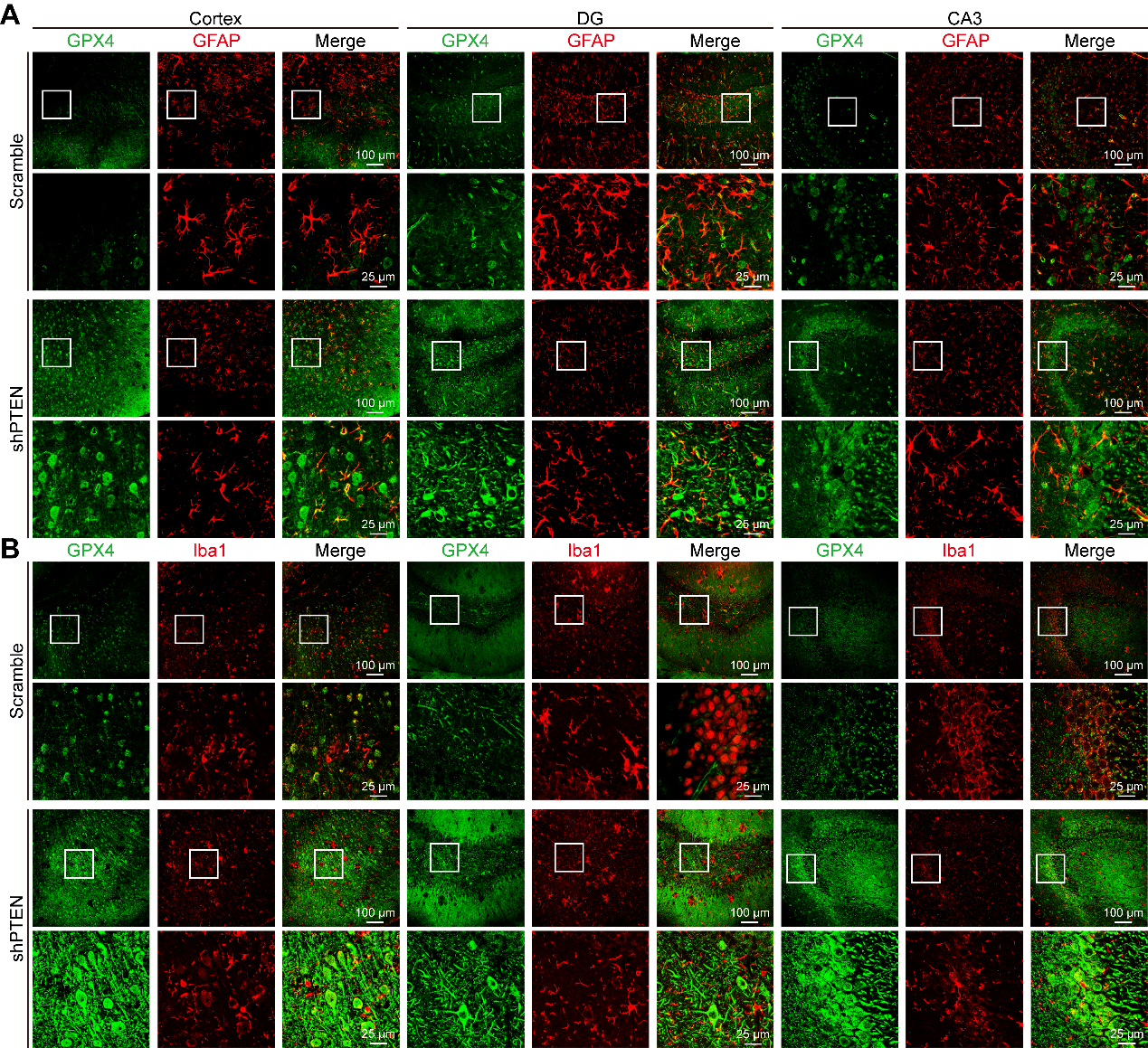


**Figure S8.** A, B) Representative immunofluorescence images of GPX4/GFAP and GPX4/Iba1 co-staining in different brain regions of APP/PS1 transgenic mice. Scale bars, 100 μm and 25 μm.


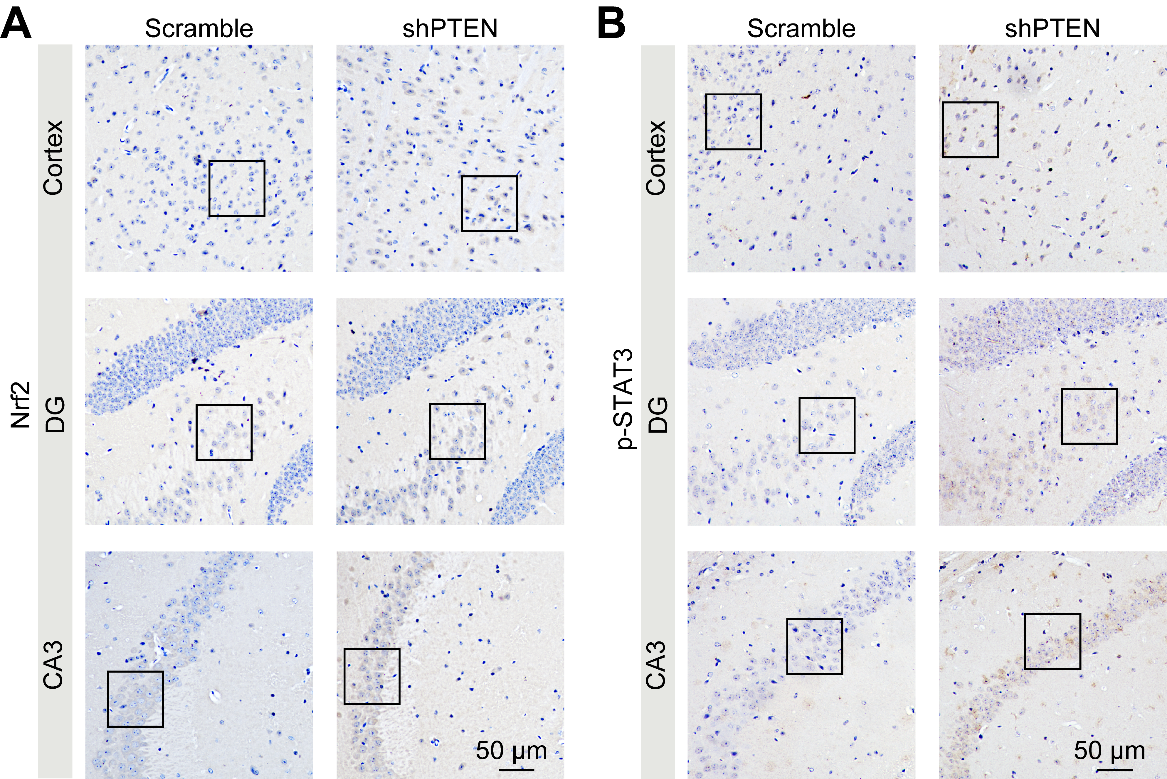


**Figure S9.** A, B) Representative images of Nrf2 and p-STAT3 immunohistochemical staining in the cortex, DG, and CA3 regions of APP/PS1 mouse brains. The black selection indicates the enlarged area and is shown in Figure 4G. Scale bar, 50 μm.

**
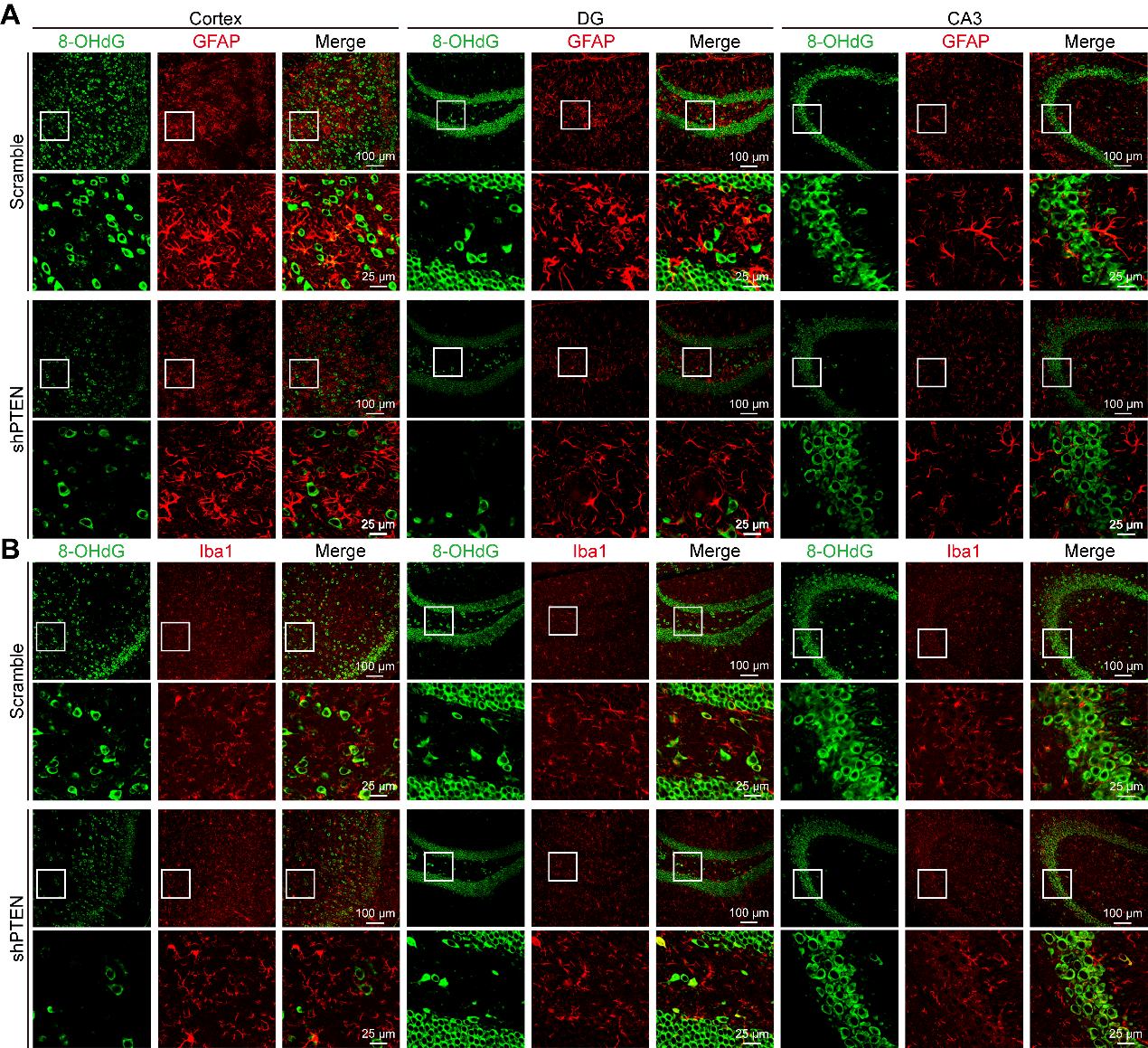
**

**Figure S10.** A, B) Representative immunofluorescence images of 8-OHdG co-staining with GFAP or Iba1 in mouse brain tissue. Scale bars, 100 μm and 25 μm.


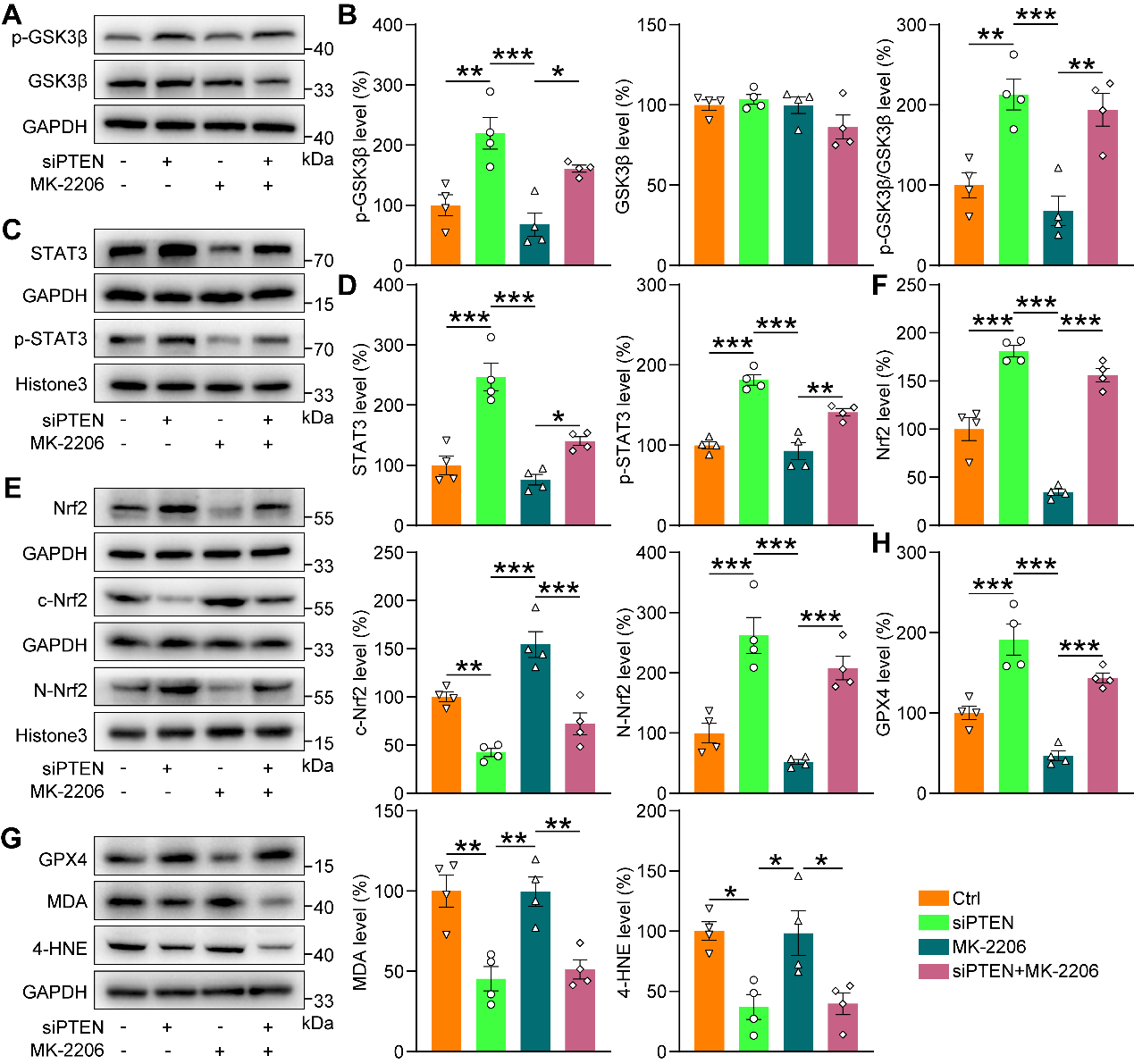


**Figure S11.** A-H) Western blot analysis of p-GSK3β, GSK3β, STAT3, p-STAT3, Nrf2, c-Nrf2, N-Nrf2, GPX4, MDA, and 4-HNE in N2a-APPsw cells under different treatments. Histone3 and GAPDH were used as nuclear and cytoplasmic loading controls, respectively. *n* = 4. **P* < 0.05, ***P* < 0.01, ****P* < 0.001.

**
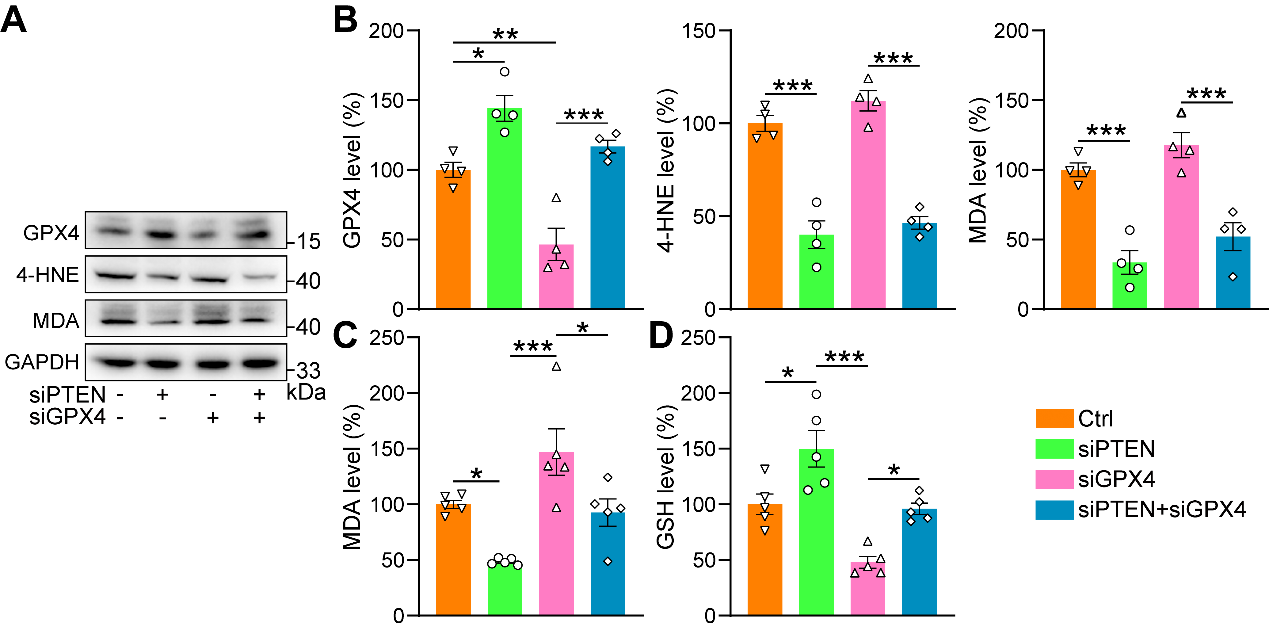
**

**Figure S12.** A, B) Western blot analysis of GPX4, 4-HNE, and MDA in N2a-APPsw cells under different treatment conditions. *n* = 4. C, D) Levels of MDA and GSH in N2a-APPsw cells measured using assay kits. *n* = 5. **P* < 0.05, ***P* < 0.01, ****P* < 0.001.


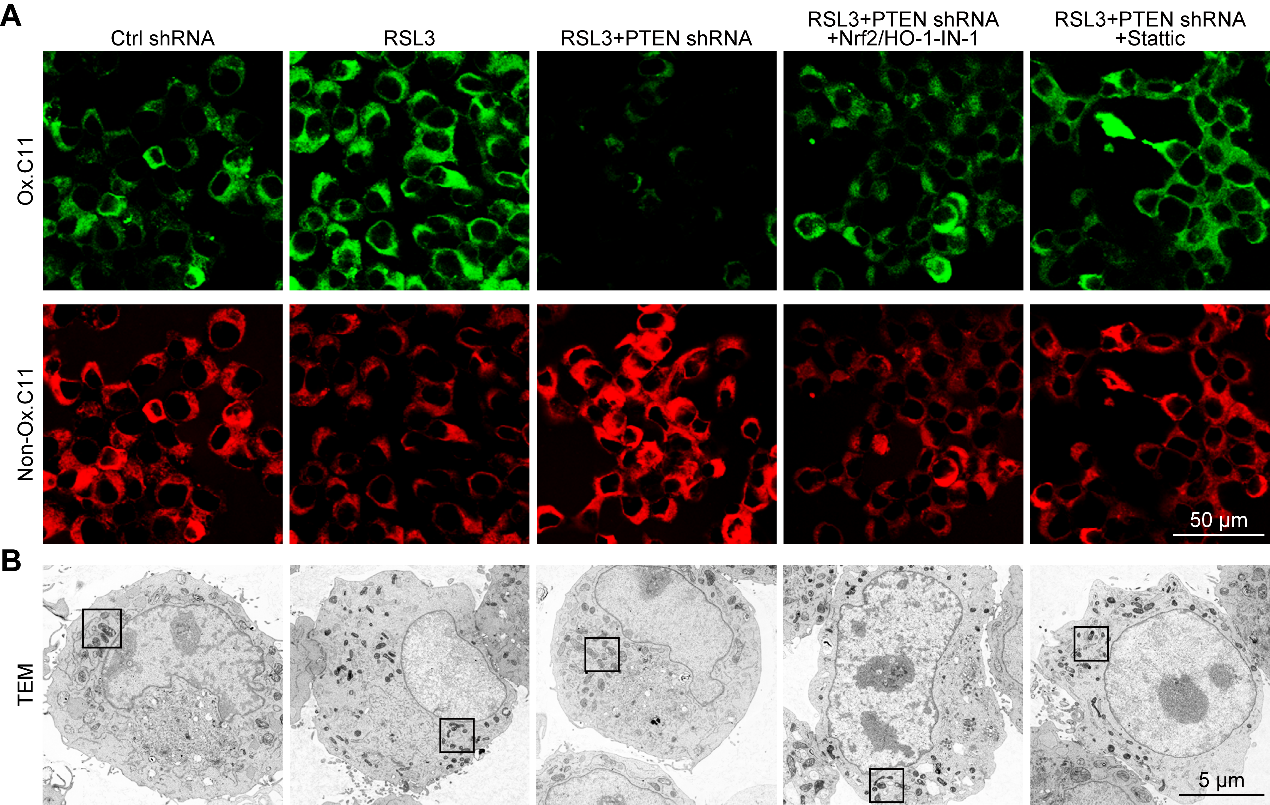


**Figure S13.** A) Representative confocal images showed lipid peroxidation changes in C11-BODIPY stained N2a-APPsw cells. Merged images are shown in Figure 7M. Scale bar, 50 μm. B) Representative TEM images of mitochondrial morphology in N2a-APPsw cells. The black selection indicates the enlarged area and is shown in Figure 7S. Scale bar, 5 μm.


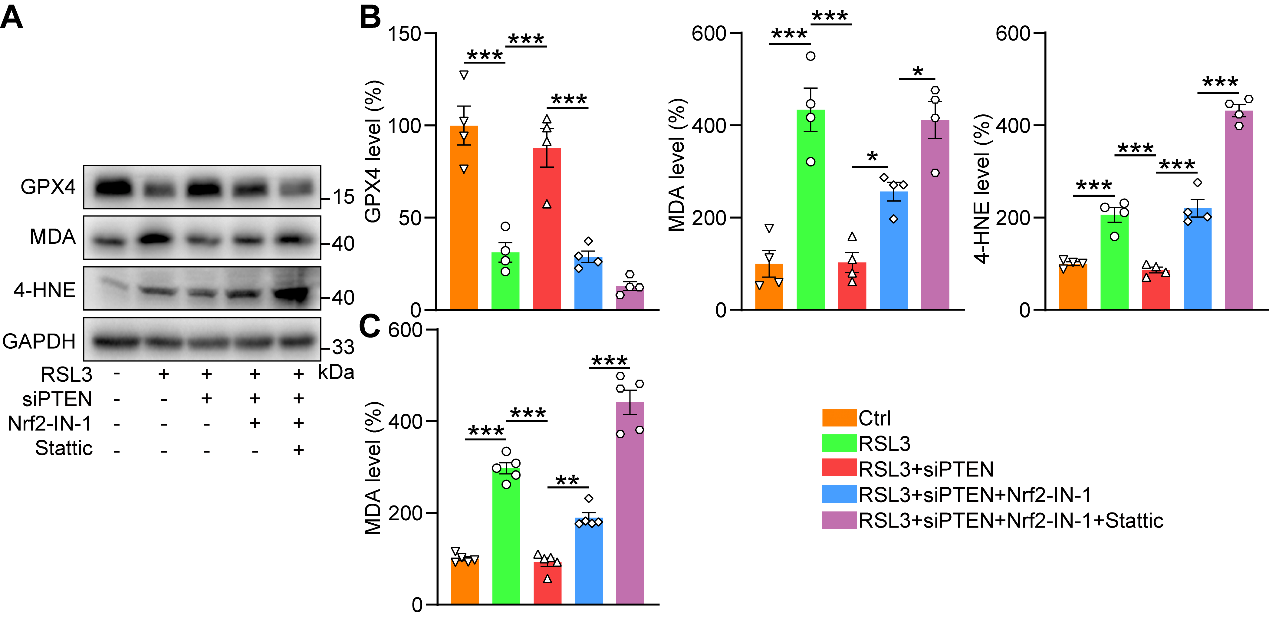


**Figure S14.** A, B) Western blot analysis of GPX4, MDA, and 4-HNE in N2a-APPsw cells under different treatment conditions. *n* = 4. C) Levels of MDA in N2a-APPsw cells measured using assay kits. *n* = 5. **P* < 0.05, ***P* < 0.01, ****P* < 0.001.


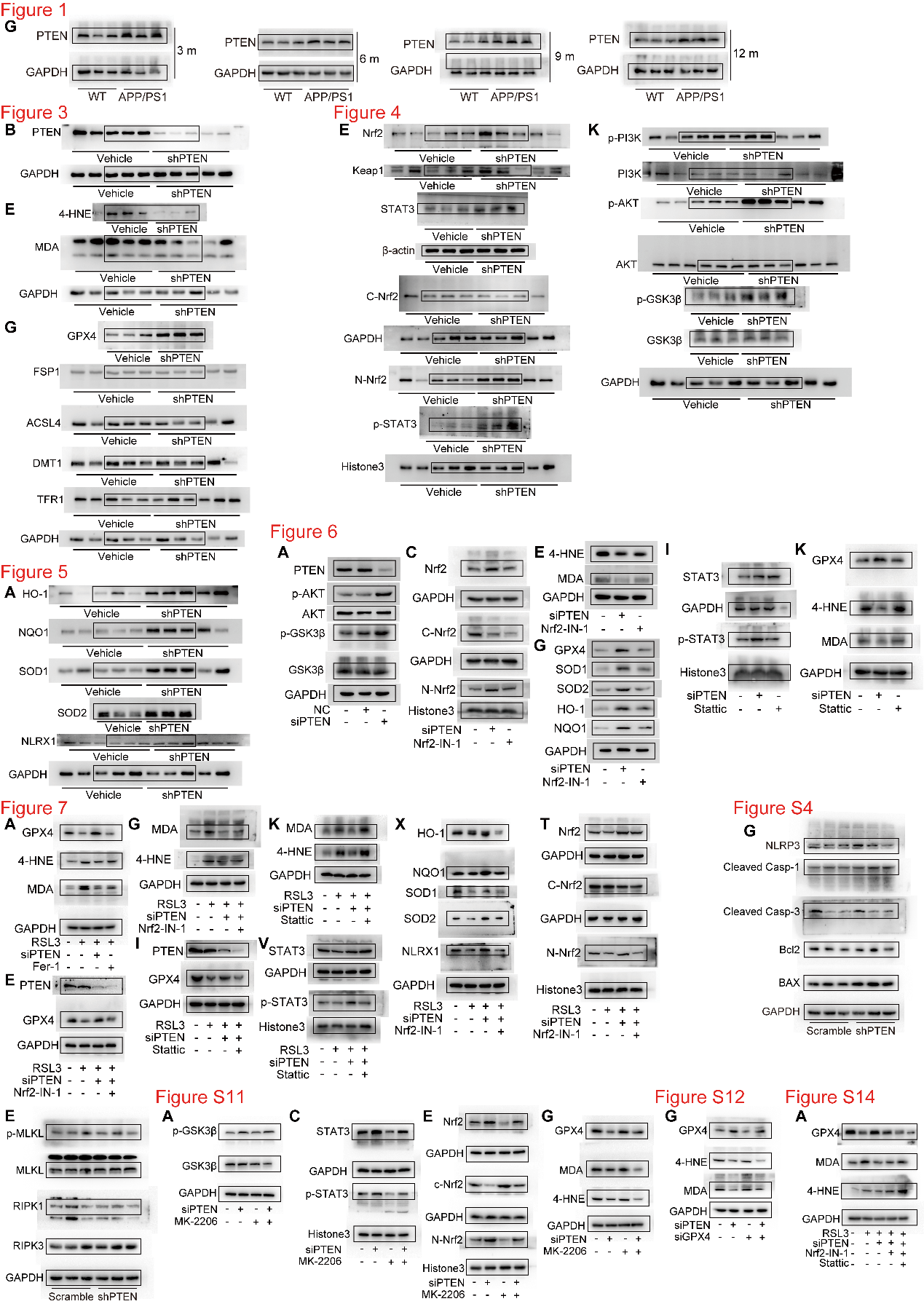


**Figure S15.** Uncropped immunoblots associated with the indicated figures.
